# L-cysteine contributes to destructive activities of odontogenic cysts/tumor

**DOI:** 10.1007/s12672-024-00959-5

**Published:** 2024-04-08

**Authors:** Ji Li, Chunyu Feng, Xiaochan Pang, Xiang Li, Xinyu Dou, Erhui Jiang, Zhengjun Shang

**Affiliations:** 1 State Key Laboratory of Oral & Maxillofacial Reconstruction and Regeneration, Key Laboratory of Oral Biomedicine Ministry of Education, Hubei Key Laboratory of Stomatology, School & Hospital of Stomatology, Wuhan University, 237 Luoyu Road, Hongshan District, Wuhan, 430079 China; 2 Department of Oral and Maxillofacial Head Neck Surgery, School & Hospital of Stomatology, Wuhan University, 237 Luoyu Road, Hongshan District, Wuhan, 430079 China

**Keywords:** Odontogenic cysts/tumor, High-throughput targeted metabolomics, L-cysteine, Cystathionine γ-lyase, Ferroptosis, Bone destruction

## Abstract

**Background:**

Odontogenic cysts/tumor can cause severe bone destruction, which affects maxillofacial function and aesthetics. Meanwhile, metabolic reprogramming is an important hallmark of diseases. Changes in metabolic flow affect all aspects of disease, especially bone-related diseases. At present, the researches on pathogenesis of odontogenic cysts/tumor are mainly focused on the level of gene regulation, but the effects of metabolic alterations on odontogenic cysts/tumor have still underexplored.

**Materials and methods:**

Imaging analysis was used to evaluate the lesion size of different odontogenic lesions. Tartrate resistant acid phosphatase (TRAP) and immunohistochemistry (IHC) assays were utilized to detect the differences in bone destruction activity in odontogenic cysts and tumors. Furthermore, metabolomics and weighted gene co-expression network analysis (WGCNA) were conducted for the metabolomic features and key metabolite screening, respectively. The effect of ferroptosis inhibition on bone destruction was confirmed by IHC, immunofluorescence, and malondialdehyde colorimetric assay.

**Results:**

The bone destruction activity of ameloblastoma (AM) was the strongest and the weakest in odontogenic cysts (OC). High-throughput targeted metabolomics was used to map the metabolomic profiles of OC, odontogenic keratocyst (OKC) and AM. WGCNA and differential analysis identified L-cysteine in OKC and AM. Cystathionine γ-lyase (CTH) was further screened by Kyoto Encyclopedia of Genes and Genomes (KEGG) analysis. The functions of L-cysteine were further validated. Finally, we confirmed that CTH affected destructive activities by regulating the sensitivity of epithelial cells to ferroptosis.

**Conclusion:**

High-throughput targeted metabolomics performed on diseased tissue confirmed the unique alteration of metabolic profiles in OKC and AM. CTH and its metabolite L-cysteine are the key factors regulating destructive activities.

**Supplementary Information:**

The online version contains supplementary material available at 10.1007/s12672-024-00959-5.

## Introduction

Odontogenic cysts/tumor are the one of the common reasons of jaw destruction. Although these diseases are benign, they often lead to huge destruction of the jaw and even pathological fracture due to delayed diagnosis and treatment and a recurrent tendency and characteristics of local invasive growth, which may pose challenges for clinical treatment, such as odontogenic keratocyst (OKC) and ameloblastoma (AM) [1]. In addition to OKC and AM, which have intense jaw destruction, the chronic and gentle expansion characteristics make odontogenic cysts (OC) also have jaw destruction ability.

The abnormal biological behaviors of OKC, including growth along the long axis of the mandible and a remarkedly high recurrence rate, attribute to its abnormal molecular events [2]. Its complex development trajectory and local infiltration lead to the difficulty of clinical diagnosis and the uncertainty of treatment. Therefore, there is an urgent need to find new diagnostic markers and treatment methods. AM is considered the most clinically common odontogenic tumor. Moreover, AM has been classified as a borderline tumor due to its local aggressiveness, high incidence and recurrence rate [3].

Metabolic reprogramming is a critical hallmark of diseases [4, 5]. Changes in metabolic flow affect all aspects of disease, especially bone-related diseases. Disordered metabolism and pathways related to metabolism promote osteoclast progression in osteosarcoma (Han et al. 2021). In jaw studies, bisphosphonates alter bone homeostasis by affecting collagen synthesis (Simon et al. 2010). With aging, dental follicles also exhibit age-related changes in metabolic profiles [6]. At the same time, changes in the external environment can also lead to changes in the metabolic profile of OKC in terms of oxidative stress and other aspects [7]. In recent years, metabolomics analysis techniques have been rapidly developed. High-throughput targeted metabolomics technology can simultaneously semi-quantitatively detect the content of a large number of metabolites [8], comprehensively analyze metabolic changes, and greatly promote the study of disease mechanisms. However, studies on the metabolic status of jaw lesions are extremely limited, even the mapping of metabolic profiles for common AM and OKC.

Cysteine metabolism is a key cellular process in which cystathionine γ-lyase (CTH) plays an irreplaceable role. CTH catalyzes the production of cysteine and hydrogen sulfide simultaneously. Hydrogen sulfide is an intracellular molecule that plays a critical regulatory role in important biological processes [9]. The amino acid cysteine is a vital metabolite in cells and plays an indispensable role in the regulation of ferroptosis, a type of programmed cell death triggered by lipid peroxidation [10, 11]. It has been reported that the potential mechanism of ferroptosis may involve the generation of glutathione (GSH) and sulfide/persulfide [10, 12] as well as depletion of cysteine. Inhibition of CTH was reported to induce ferroptosis in cells [11, 13].

In this study, we first mapped the metabolic profiles among different odontogenic cysts/tumor by high-throughput targeted metabolomics, and then found the representative metabolic modules of each disease by WGCNA. Subsequently, the key differential metabolites were explored in the feature module. We further selected cysteine, which is related to bone destruction, for further study. Further, we investigated the role of CTH and its by-product hydrogen sulfide in regulating cysteine metabolism and promoting local invasion of OKC and AM. Our work also revealed that the abnormal metabolism of cysteine, facilitated by CTH, conferred cellular protection against ferroptosis. The hydrogen sulfide generated from cysteine metabolism stimulated the NF-κB signaling pathway and the activation of nitrogen fixation 1 homolog (NSF-1), leading to increased expression of cathepsin K (CTSK), matrix metalloproteinases 9 (MMP9) and ferroptosis tolerance, thereby enhancing local invasion and cell viability of OKC and AM. Overall, our current study has identified CTH and its role in abnormal cysteine metabolism as potential therapeutic modalities for intervening in the local invasion and growth of OKC and AM.

In conclusion, our study contributes to the understanding of the overall etiology and the similarities and differences of various odontogenic cysts/tumor. As the first high-throughput targeted metabolomic study of odontogenic cysts/tumor, we innovatively report the metabolic disorder patterns and key metabolites in patients with odontogenic cysts/tumor.

## Material and methods

### Specimen collection

37 specimens with benign lesions of the jaw were collected by senior clinician with extensive clinical experience from Hospital of Stomatology, Wuhan University for subsequent experiments. The pathological diagnosis of all patients was confirmed by two senior pathologists with more than 10 years of clinical experience. Our study was approved by the Ethics Committee of School & Hospital Stomatology, Wuhan University (IRB-ID: 2021B54). All patients included in the study agreed to be included in the study by written informed consent. The demographic data of the relevant patients are detailed in the attached table.

### Data acquisition

RNA-seq data and clinical data were downloaded from GEO database (GSE38494). GSEA software (4.1.0) was used for gene set enrichment analysis (GSEA). The R package “clusterProfiler” was used to perform the Kyoto Encyclopedia of Genes and Genomes (KEGG) analysis in R software (version 4.0.2).

### Imaging analysis

As described before, formulas: $$\frac{4\pi }{3}*\frac{a*b*c}{2}$$ can be used to estimate the extent of bone destruction according to the radiograph images [16].

### Tartrate resistant acid phosphatase (TRAP) assay

Experiments were performed using the TRAP kit (Beyotime, China) according to the instructions of reagent manufacturer. In brief, surgical specimens obtained were immediately rinsed 3 times with precooled deionized water followed by flash freezing in liquid nitrogen for 15 min. Subsequently, the samples were briefly stored at – 80 ℃. 100 mg tissues were individually grounded with liquid nitrogen and the homogenate was resuspended with Pierce™ IP lysate buffer (Thermo Fisher Scientific, USA). The samples were stood on ice for 5 min. Supernatants were collected by centrifugation (4 ℃, 12000 g, 15 min) after lysis of benign lesions and cells for subsequent experiments. After mixing with chromogenic substrate and tartaric acid solution, the mixture was incubated at 37 ℃ for 10 min. The absorbance was measured at 405 nm after terminating the reaction with the termination solution.

### Immunohistochemistry (IHC)

For the IHC staining, surgical specimens from patients were fixed with 10% formalin and embedded in paraffin. After dewaxing and rehydration, endogenous peroxidase was inactivated, enclosed and incubated with the corresponding primary, secondary and tertiary antibodies. The DAB process was then used for color development. For the IHC statistical analyses, the process was carried out as described in our previous study [14]. The final score (H score) was used as an indicator of IHC staining intensity.

### High throughput targeted metabolomics by liquid chromatography-mass spectrometry (LC–MS)

Metabolic profiling performed in the present study included sample preparation, metabolite extraction, and LC_MS analysis. Sample preparation was performed according to the requirements of the relevant companies. In brief, surgical specimens obtained were immediately rinsed three times with precooled deionized water followed by flash freezing in liquid nitrogen for 15 min. Subsequently, the samples were briefly stored at − 80 ℃. 100 mg tissues were individually grounded with liquid nitrogen and the homogenate was resuspended with prechilled 80% methanol. The samples were stood on ice for 5 min and then centrifuged (4 °C, 15,000 g, 20 min). Supernatant was diluted to final concentration containing 53% methanol by LC–MS grade water. The samples were centrifuged again (4 °C, 15,000 g, 20 min). Finally, the supernatant was injected into the Triple quadrupole–linear ion trap composite SCIEX QTRAP^®^ 6500 + mass spectrometer. The detection of the experimental samples using Multiple Reaction Monitoring (MRM) were based on novogene in-house database. The Q3 were used to the metabolite quantification. The Q1, Q3, retention time (RT), declustering potential (DP) and collision energy (CE) were used to explicit the metabolite. The data files were processed using the SCIEX OS (version 1.4) to integrate and correct the peak. The main parameters were set as follows: minimum peak height: 500; signal/noise ratio: 5; and gaussian smooth width: 1. The area of each peakrepresents the relative content of the corresponding substance. A total of 37 lesion samples and 5 quality control (QC) samples were used for metabolomic analysis by. For tissue metabolomics analysis, the QC sample was tested before the analytical run and was frequently injected once every ten samples throughout the analytical run to monitor instrument stability. The resulting data are standardized for downstream analysis.

### WGCNA

WGCNA was used to analyze the normalized metabolomic data in R software (version 4.0.2). Firstly, the hierarchical clustering method was used to find the outlier samples, and it was found that all samples were non-outlier samples. A R2 of 0.9 and a soft threshold of 4 were chosen for subsequent analysis. Then, the adjacency matrix is transformed into topological overlap matrix (TOM). Hierarchical clustering was used to identify modules (minModuleSize = 10, mergeCutHeight = 0.25). Feature genes were calculated and hierarchical clustering was performed for each module. Module characteristic gene (ME) and module membership (MM) were used to find important modules. Subsequently, by calculating the Pearson correlation coefficient between each module and each disease, the module with the strongest correlation was selected for further analysis. The selection of genes highly related to the disease was continued in each disease representative module. Finally, we combined the representative metabolites of each disease with the results of differential analysis to select the downstream study metabolites.

### Cells and cell culture

The Human immortalized epidermal cells HaCaT was obtained from the Shanghai Institute of Cell Biology, Chinese Academy of Sciences (Shanghai, China) and cultured in DMEM (Hyclone, USA) with high glucose with 10% fetal bovine serum at 37 °C with 5% CO2.

### Cell transfection

For the cell transfection, the process was carried out as described in our previous study [15]. Lipofectamine^™^ 3000 (Invitrogen, USA) was used to transfect small interfering RNA (siRNA). The targeting sequence of siRNAs (GenePharma, China) against human cystathionine γ-lyase (CTH) was 5′- GGAGCUGAUAUUUCUAUGU-3′ (#1) and 5′- CCUGGUGUCUGUUAAUUGU-3′ (#2). The transfection efficiency was identified by Western Blot.

### Western blot

Cells were lysed using mammalian protein extraction reagent (Beyotime, China) with protease inhibitor (ROCHE, Switzerland) and phosphatase inhibitor (ROCHE, Switzerland). Subsequently, protein concentration was determined by bicinchoninic acid protein assay kit (Beyotime, China). Next, 5 × SDS loading buffer (Beyotime, China) was added into the protein solutions and heated for 15 min at 95 °C. Then, 15 mg of protein was used for electrophoresis (60 V, 30 min; 110 V, 70 min) and transfer (200 mA, 120 min). Subsequently, blocking using QuickBlock™ Western blocking buffer (Beyotime, China) and antibody incubation were performed. The primary and secondary antibodies used are shown in the Supplementary Table 1.

### Malondialdehyde (MDA) colorimetric assay

According to the manufacturer's instructions, MDA concentration in cultured cells of indicated treatments was measured by an MDA assay kit (Elabscience, China) Briefly, MDA in the sample reacts with thiobarbituric acid (TBA) to generate an MDA-TBA adduct. The MDA-TBA adduct can be quantified colorimetrically (OD = 532 nm). The relative concentration of MDA in the samples was calculated as fluorescence intensity per milligram of protein. All assays were carried out in triplicates and repeated at least three times.

### GSH enzyme-linked immunosorbent assay

Intracellular glutathione level in samples or cultured cells of indicated treatments was determined using the GSH enzyme-linked immunosorbent assay kit (CAS: E-EL-0026c, Elabscience, Hubei, China) in accordance with the manufacturer’s instructions. All assays were carried out in triplicates and repeated at least 3 times.

### Intracellular ferrous iron assay

Intracellular ferrous iron level in cultured cells of indicated treatments was determined using the cell ferrous iron colorimetric assay kit (CAS: E-BC- K881-M, Elabscience, Hubei, China) in accordance with the manufacturer’s instructions. Briefly, Ferrous ions were combined with the probe supplied by the kit and The Ferrous ions -probe adduct can be quantified colorimetrically (OD = 600 nm). The relative level of ferrous iron in the samples was calculated as fluorescence intensity per milligram of protein.

### Cysteine, pyruvate and hydrogen sulfide synthesis assay

Intracellular cysteine, pyruvate and hydrogen sulfide level in cultured cells of indicated treatments was determined using the cysteine assay kit (CAS: E-BC- K352-M, Elabscience, Hubei, China), pyruvic acid colorimetric assay kit (CAS: E-BC- K130-M, Elabscience, Hubei, China) and hydrogen sulfide colorimetric assay kit (CAS: E-BC- K355-M, Elabscience, Hubei, China) in accordance with the manufacturer’s instructions.

### Statistical analysis

All experiments were performed in triplicate and repeated at least 3 times. Shapiro–Wilk and Kolmogorov–Smirnov tests were used to analyze the data for normal distribution. Data were expressed as mean ± SD and analyzed by analysis of variance (ANOVA) and Student’s t test by the Prism (version 6.0) and R software (version 4.0.2). Difference was considered significant when P < 0.05.

## Result

### Clinical features of odontogenic cysts/tumor

**Fig. 1:**
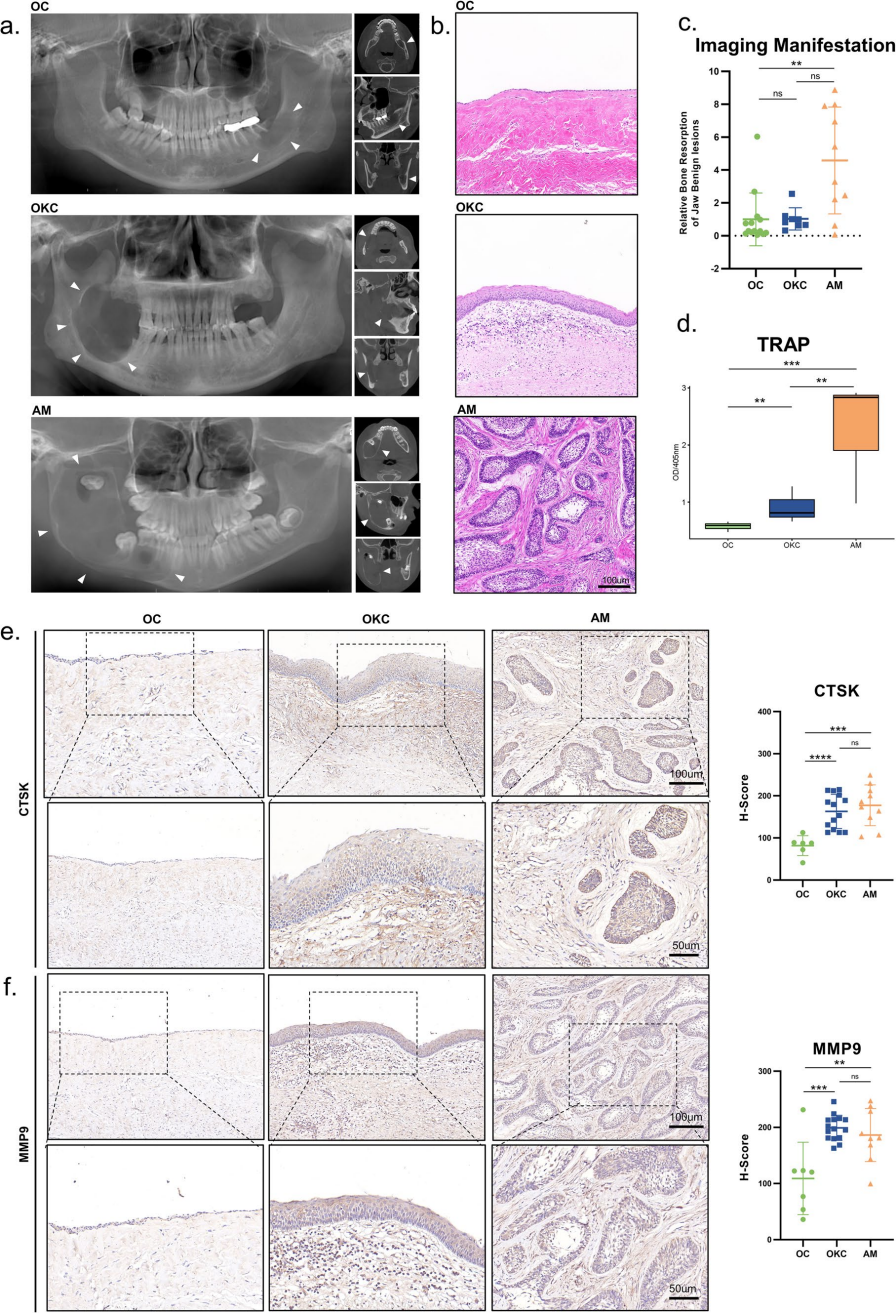
Differences in the extent of bone destruction in odontogenic cysts/tumor. **a** Panoramic radiographs in OC, OKC and AM. **b** H&E stain of OC, OKC and AM. **c** Statistical plot of the extent of bone destruction. **d** TRAP stain of OC, OKC and AM. **e** CTSK expression of OC, OKC and AM tissues. **f** MMP9 expression of OC, OKC and AM. Data information: All data shown (**c**–**f**) represent the means _ SD (n ≥ 3 biological replicates). ANOVA followed by Tukey’s post hoc test (**c**–**f**) was used for the statistical analysis. (*P < 0.05; **P < 0.01; ***P < 0.001)

The specimens in this study included OC, AM, and OKC. Representative imaging data and pathological data are shown in the Fig. 1a, b. We compared the pattern of bone destruction shown on radiographs, according to the result, it was confirmed that AM had the largest extent of destruction, and the unique pattern of destructive activities resulted in the strongest destructive capacity of AM (Fig. 1c). The unique pattern of destructive activities resulted in the strongest destructive capacity of AM (Fig. 1c). TRAP assay is commonly used to detect bone destruction capacity. We confirmed that AM had the strongest capacity for bone destruction, followed by OKC and OC the least (Fig. 1d). Both CTSK and MMP9 can achieve bone destruction through tissue matrix remodeling, and their high expression often predicts strong bone destruction capacity [17]. The IHC also confirmed that the bone destruction capacity of AM and OKC was significantly higher than that of OC. Among them, AM had the highest expression of CTSK (Fig. 1e), while MMP9 was the strongest in OKCs (Fig. 1f). The large deposition of Col1A1 is associated with tissue repair [18]. In Supplementary Fig. 1a, we also observed that AM had a lower expression of Col1A1.

### Metabolic differences among odontogenic cysts/tumor

**Fig. 2:**
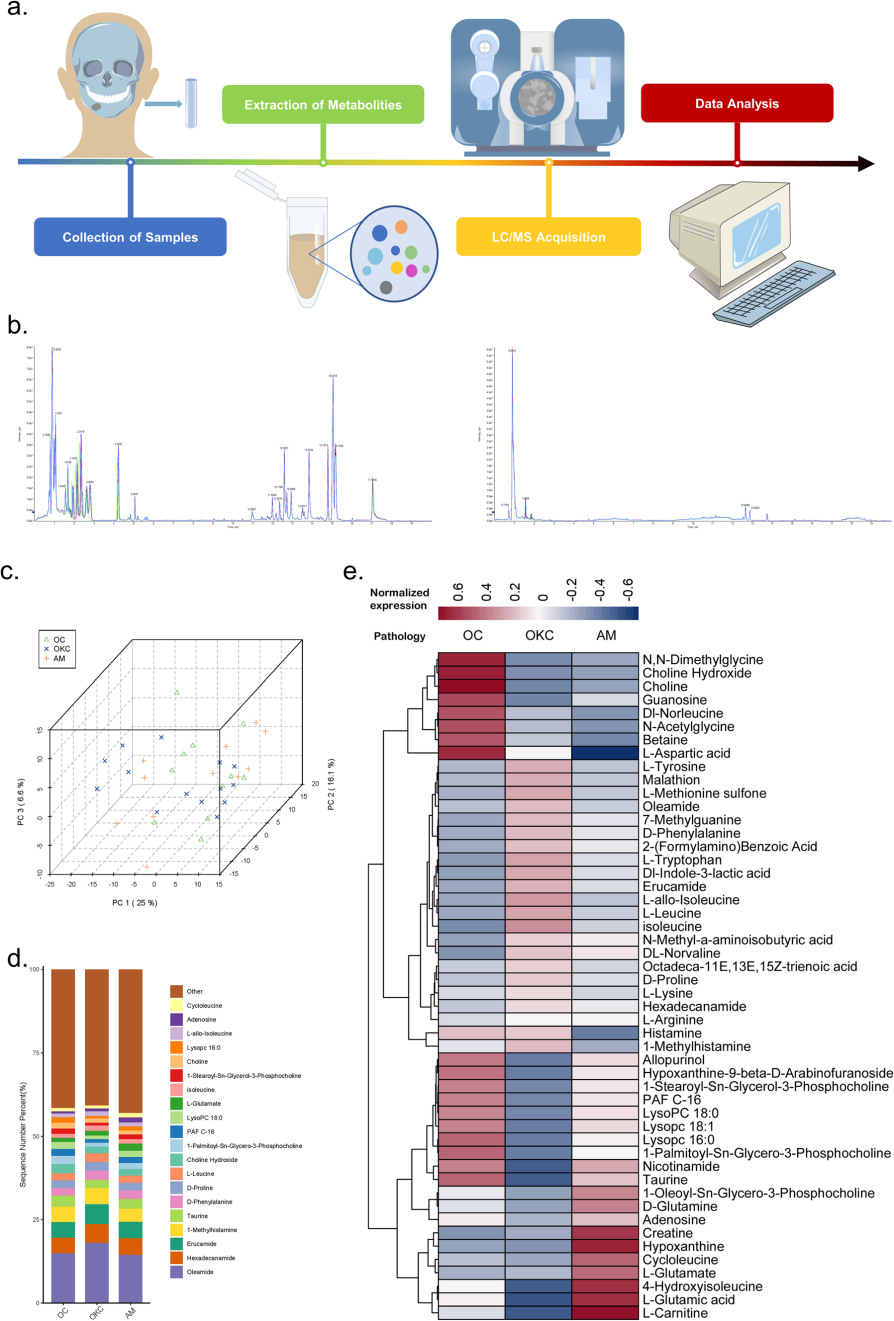
Metabolic profiling of odontogenic cysts/tumor. **a** High-throughput targeted metabolome flow diagrams. **b** The chemical composition of the metabolites was analyzed and representative total ion chromatograms (TIC) in positive and negative ion modes. **c** PCA of OC, OKC and AM. **d** metabolite composition of OC, OKC and AM. **e** Cluster heat map analysis of OC, OKC and AM

As shown in Fig. 2a, we performed metabolic profiling by employing the LC–MS/MS method and standardized the obtained data for subsequent analysis. 37 patients were included in this study, and the demographic and clinical characteristics are shown in Supplementary Table 2. A total of 697 metabolites were identified by high-throughput targeted metabolomics assays in tissue samples. As usual [19], the typical total ion chromatograms (TIC) map was obtained and the chemical composition of the metabolites was analyzed and representative TIC map in positive and negative ion modes were shown in Fig. 2b. The metabolites were identified (Supplementary Fig. 1a) and quality control was performe (Supplementary Fig. 1b). First, unsupervised principal component analysis (PCA) was performed after data standardization and showed significant clustering of OC, OKC, and AM tissues (Fig. 2c). Principal component (PC) 1 accounted for 25% of the variance observed among the metabolites associated with the three diseases. The initial three PCs collectively elucidated 47.7% of the variation among these factors. Subsequently, we found differences among the constituent components of each group by PCA (Supplementary Fig. 1c, d, e,). We then showed differences in metabolite composition among different groups (Top 20 were listed) (Fig. 2d). Among the three diseases, OC exhibited the highest lipids, while AM showcased the highest nucleic acid levels, and OKC displayed the most abundant peptides content. And then, the top 50 metabolites of relative abundance were selected for sample clustering (Fig. 2e). We found different metabolite enrichment patterns in different diseases. Examples include N, N-Dimethylglycine and Choline hydroxide, which are highly enriched in OC, Isoleucine and L-Methionine sulfone in OKC, and L-Garnitine and Hypoxanthine in AM. Taken together, the metabolic profile suggests a unique metabolic reprogramming of OKC and AM.

Further, we explored the differences of metabolic profiles. Firstly, orthogonal partial least squares discriminant analysis (oPLS-DA) showed a clear separation between AM and OC (R2X = 0.332, R2Y = 0.984, Q2 = 0.494) (Supplementary Fig. 2a). The validity of the oPLSDA model was confirmed by permutation tests of 100 interactions (Supplementary Fig. 2b). Differential expression analysis identified significant differences in 13 metabolites, of which 8 metabolites were depleted and 5 metabolites were increased (Supplementary Fig. 2c, Supplementary Table 2). The top 5 metabolites of AM metabolism changes were the Nicotinamide D-ribonucleotide, L-Cysteine, L-Cystathionine, Carnosine and S-Sulfo-L-cysteine. Based on the differential metabolites, we identified 9 differential metabolic pathways in AM, including taurine and hypotaurine metabolism, nicotinate-nicotinamide metabolism, and glycine, serine and threonine metabolism (Supplementary Fig. 2d, Supplementary Table 3).

Similarly, oPLS-DA with permutation test confirmed the difference between OKC and OC (R2X = 0.361, R2Y = 0.968, Q2 = 0.573) (Supplementary Fig. 2e, & f). And subsequently, results showed that 6 metabolites decreased and 4 metabolites increased in OKC (Supplementary Fig. 2 g, Supplementary Table 4). Based on the differential metabolites of Diphosphoric acid, Nicotinamide D-ribonucleotide, Urocanate, Propane-1,2,3-tricarboxylate, and other metabolites, we found 10 differential metabolic pathways, including citrate cycle, pyrimidine metabolism, and galactose metabolism (Supplementary Fig. 2 h, Supplementary Table 5).

Inflammatory response has a certain impact on metabolism (He et al. 2020), and metabolic differences exist between healthy pulp and periapical lesions (Altaie et al. 2021). Therefore, we aimed to explore the metabolic compositions of NOC and IOC. The significantly clustered NOC and IOC also had different metabolite compositions, suggesting distinct metabolic processes between the two types of lesions (Supplementary Fig. 3a, b, and c). 8 metabolites were absent and 14 metabolites (Supplementary Fig. 3f) were increased in the IOC group with significant differences showed by oPLS-DA (R2X = 0.38, R2Y = 0.938, Q2 = 0.209) (Supplementary Fig. 3d, e, Supplementary Table 6). Histidine metabolism, primary bile acid biosynthesis, caffeine metabolism caused by vitamin C, hydantoin-5-propionate, glycocholate, glycochenodeoxycho-late and taurochenodeoxycholate were significantly different metabolic pathways in IOC (Supplementary Fig. 3f, and g, Supplementary Table 7). The same approach was used in the difference analysis between non- inflammatory OKC (NOKC) and inflammatory OKC (IOKC). The initial three PCs collectively elucidated 59.2% of the variation among these factors (Supplementary Fig. 4a). Different metabolites were shown in Supplementary Fig. 4b, and c. Similarly, PCA and permutation test were performed in NOKC and IOKC (Supplementary Fig. 2d, & e). And subsequently, results showed that 8 metabolites decreased and 13 metabolites increased in IOKC (Supplementary Fig. 4f, Supplementary Table 7). Based on the differential metabolites, we found 8 differential metabolic pathways (Supplementary Fig. 4 g, Supplementary Table 8).

### WGCNA and differential analysis confirmed L-cysteine as the characteristic metabolite of OKC

**Fig. 3:**
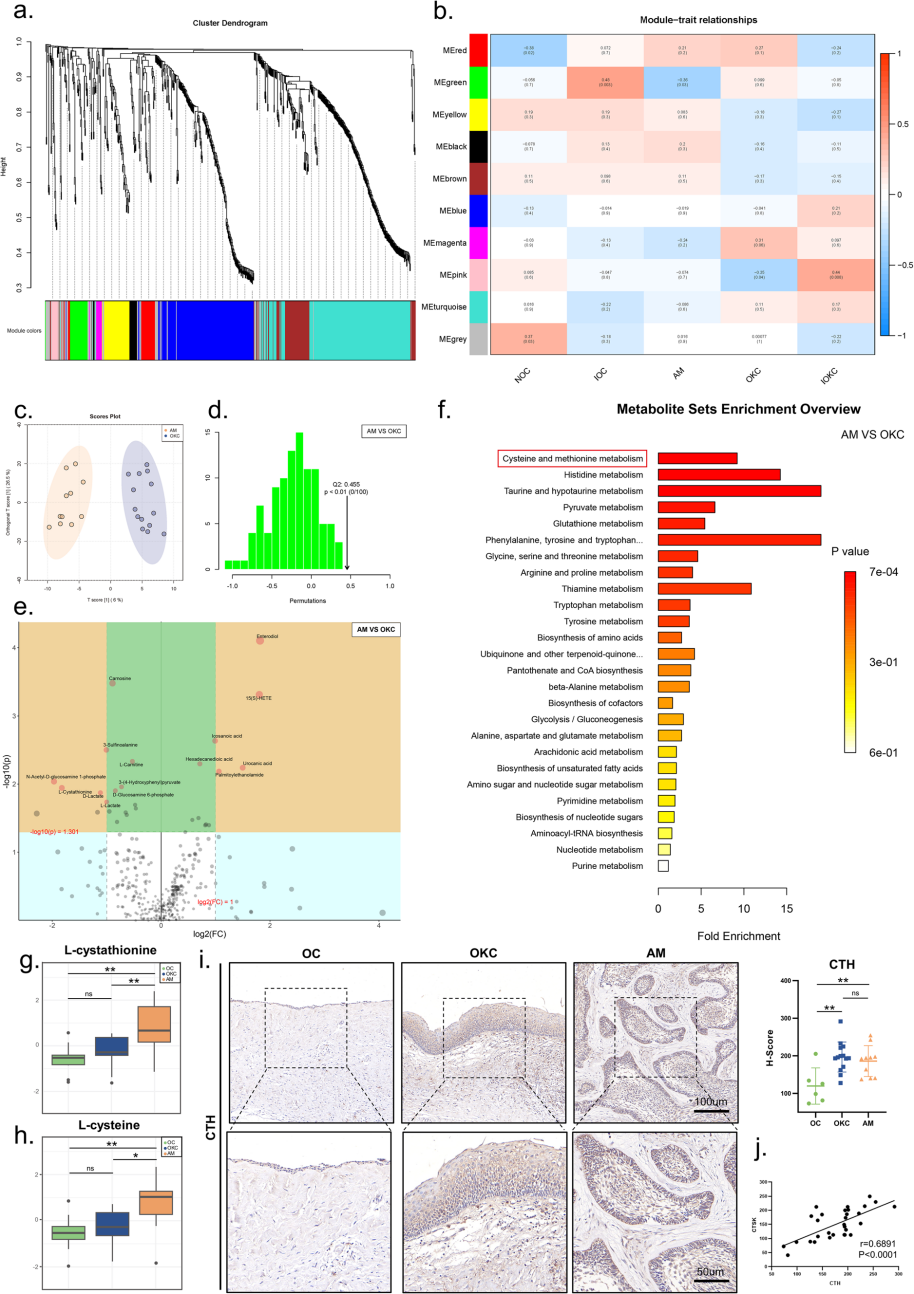
WGCNA and differential analysis confirmed L-cysteine as the characteristic metabolite of OKC. Cluster tree analysis **a** and module analysis **b** among OC, OKC and AM. **c** PCA of AM and OKC. **d** Substitution test of AM and OKC. **e** Differential metabolite analysis of AM and OKC. **f** KEGG analysis of AM and OKC. L-cystathionine **g** and L-cysteine **h** analysis of OC, OKC and AM. **i** CTH expression of OC, OKC and AM. **j** Correlation analysis of CTH and CTSK. Data information: All data shown (**g**–**i**) represent the means _ SD (n ≥ 3 biological replicates). ANOVA followed by Tukey’s post hoc test (**g**–**i**) was used for the statistical analysis. (*P < 0.05; **P < 0.01; ***P < 0.001)

**Fig. 4:**
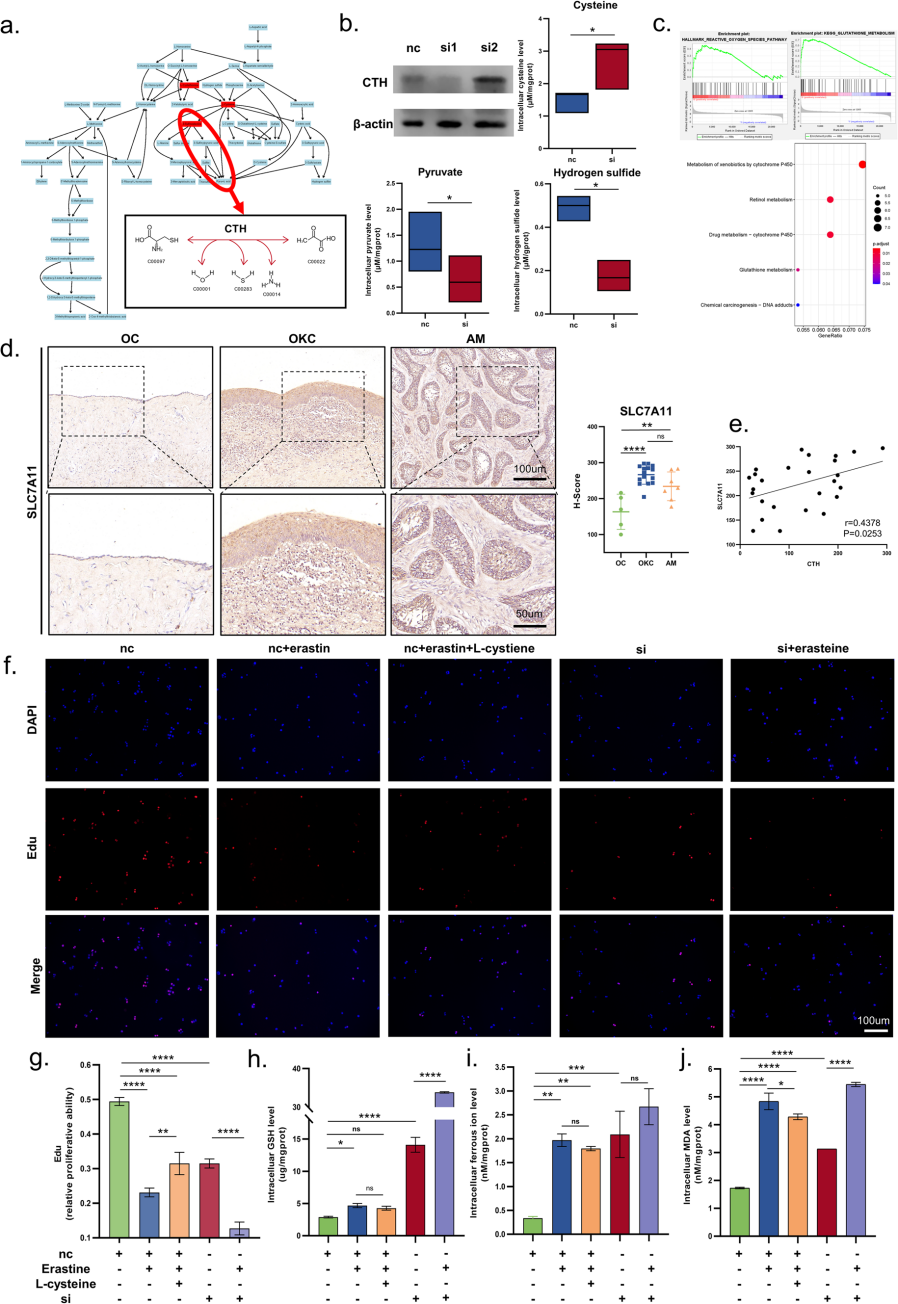
L-cysteine induced ferroptosis tolerance is a key factor in destructive activities in odontogenic cysts/tumor. **a** KEGG map of L-cysteine pathway. **b** Western Blot of HaCaT after si-CTH and pathway metabolite difference after treatment of si-CTH. **c** GSEA and KEGG analysis of OKC and AM. **d** SLC7A11 expression of OC, OKC and AM tissues. **e** Correlation analysis of CTH and SLC7A11. EdU stain (**f**) and statistical chart (**g**) of HaCaT after treatment. GSH (**h**), MDA (**i**), intracellular ferrous ions (**j**) of HaCaT after treatment. Data information: All data shown (**b**, **d**, and **f**–**j**) represent the means _ SD (n ≥ 3 biological replicates). Student’s t-test (**b**) and ANOVA followed by Tukey’s post hoc test (**d**, and **f**–**j**) was used for the statistical analysis. (*P < 0.05; **P < 0.01; ***P < 0.001)

WGCNA was used to seek the main representative modules for each group. Firstly, we searched for the outlier metabolites in the metabolites by hierarchical clustering method, and finally we found that all the metabolites were not outliers (Supplementary Fig. 5a). Using the WGCNA method, we divided the metabolites into 10 modules (Fig. 3a, b). Considering the effect of chronic inflammation on destructive activities, we divided OC into non- inflammatory OC (NOC) and inflammatory OC (IOC). Among them, NOC has 7 samples and IOC has 9 samples. The MEgreen is negatively correlated with AM (R = − 0.36, p = 0.03). The MEpink is negatively correlated with OKC (R = − 0.35, p = 0.04). As a result, the red module containing 35 metabolites became the representative module of AM (Supplementary Fig. 5b & Supplementary Table 8), and the pink module containing 13 metabolites was used for OKC (Supplementary Fig. 5c). Then, differential metabolites in each module were screened (Supplementary Table 9). We found that cysteine might be an important metabolite contributing to the development of AM, and Uridine diphosphate (UDP) played the similar role in OKC. In addition, the relationships between cysteine and AM, OKC and UDP were investigated. Similar to the aforementioned process, the obviously divergent AM and OKC had 29 differential metabolites (R2X = 0.396, R2Y = 0.944, Q2 = 0.455) (Fig. 3c, d). 12 of these metabolites were decreased and 17 metabolites were increased (Fig. 3e & Supplementary Table 8). In the comparison between OKC and AM, cysteine and methionine metabolism were the most significantly different pathways (Fig. 3f). In addition, up-regulation of 3-Sulfinoalanine, N-Acetyl-D-glucosamine 1-phosphate, D-Lactate, D-2-Aminobutyrate, L-Cysteine, and L-Cystathionine and down-regulation of Enterodiol, 15(S)-HETE, Urocanic acid, Palmitoylethanolamide, and Tricarballylate were also observed in the AM tissues. Meanwhile, the contents of cystine (Fig. 3g) and cysteine (Fig. 3h) gradually increased in OC, OKC and AM groups. According to the KEGG analysis of cysteine and methionine metabolism in OKC and AM, we traced back to CTH (Fig. 4a). Meanwhile, CTH-regulated cysteine metabolism played an essential role in the progression of OKC and AM. The difference in cysteine metabolic flux may lead to differences in biological properties between the two diseases. The expressions of CTH in the OKC, AM and OC were detected by IHC. As shown in the Fig. 3I, CTH also showed a similar trend as cysteine, which showed a gradually increase trend in OC, OKC and AM. Furthermore, the expression of CTH was positively correlated with the expression of CTSK (Fig. 3j). Thus, we hypothesized that the cysteine metabolism regulated by the CTH was the possible reason of different invasion ability of OC, OKC, and AM.

### L-cysteine induced ferroptosis tolerance is a key factor in destructive activities of odontogenic cysts/tumor

According to the KEGG pathway, the hydrogen sulfide may be produced during the process of catalyzing cysteine to the pyruvate (Fig. 4a). As a signaling molecule, hydrogen sulfide may play a role in downstream biological events. Previous studies have shown that CTH have a strong relationship with ferroptosis [20]. Thus, we further explored the reason of jaw destruction caused by CTH. After the silence of CTH in HaCaT cells, the production of hydrogen sulfide and the pyruvate was reduced, and the cysteine was accumulated (Fig. 4b). Figure 4c showed a higher expression of genes related to reactive oxygen species (ROS) and an increased GSH metabolism in the OKC and AM tissues via GSEA and KEGG. These findings imply a higher oxidative homeostasis in OKC. Further, IHC results of OKC and AM tissues displayed elevated expressions of SLC7A11 (Fig. 4d), which were related to the glutathione metabolism and ferroptosis (Fig. 4e). Both the applications of erastine and the silence of CTH in HaCaT cells resulted in the low viability of cells (Fig. 4f, g). Also, it was found that the GSH was increased when the erastine was applied and CTH was silenced (Fig. 4h). And lipid peroxidation, a hallmark of ferroptosis, was increased in cells (Fig. 4i). At the same time, the intracellular ferrous iron was accumulated (Fig. 4j). After the silence of CTH, the cells became more susceptible to the erastine (Fig. 4h–j). Next, we verified the role of cysteine in ferroptosis. As expected, exogenous L-cysteine could rescue the viability of cells and intracellular levels of GSH, lipid peroxidation and ferrous iron induced by erastin (Fig. 4h–j). To confirm that the cell death caused by CTH or the cysteine metabolism was ferroptosis, the cell apoptosis assay was conducted, the results showed that the cell death induced by absence of CTH was not apoptosis (Supplementary Fig. 5d), necrosis and autophagy (Supplementary Fig. 5e and f). Our findings confirmed that the demise of HaCaT cell line induced by CTH silencing was due to ferroptosis. In conclusion, cysteine metabolism regulated by the high expression of CTH might efficiently promote the resistance of ferroptosis in OKC and AM.

### NF-κB pathway is involved in L-cysteine-induced ferroptosis tolerance

**Fig. 5:**
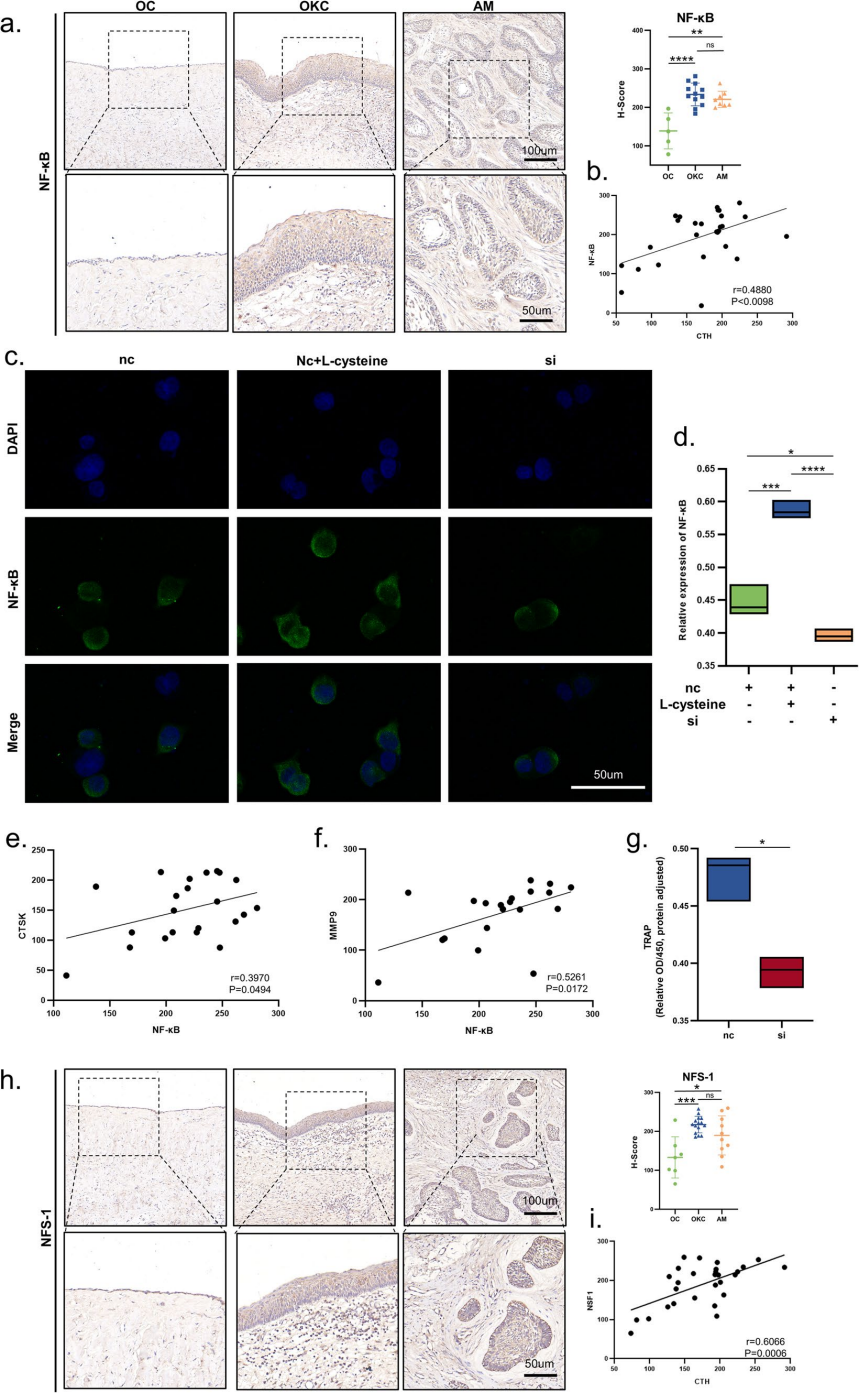
L-cysteine induced ferroptosis tolerance is associated with NF-κb pathway. (**a**) NF-κB expression among OC, OKC and AM. (**b**) Correlation analysis of NF-κB and CTH. Fluorescence (**c**) and statistical graphs (**d**) Nuclear localization of NF-κB. Correlation analysis of NF-κB and CTSK (e) and MMP9 (**f**). (**g**) TRAP of HaCaT after si-CTH. (**h**) NSF-1 expression among OC, OKC and AM. (**i**) Correlation analysis of NFS-1 and CTH. Data information: All data shown (**a**, **c**, **d**, **g** and **h**) represent the means _ SD (n ≥ 3 biological replicates). Student’s t-test (**g**) and ANOVA followed by Tukey’s post hoc test (**a**, **c**, **d** and **h**) was used for the statistical analysis. (*P < 0.05; **P < 0.01; ***P < 0.001)

At first, we found that the expression level of NF-κB was significantly increased in OKC and AM (Fig. 5a). There is also a certain correlation between the content of L-cysteine and the expression of NF-κB (Fig. 5b). The immunofluorescence staining displayed that the expression and nuclear translocation of NF-κB were increased after treated by L-cysteine (Fig. 5c, d). But after silencing CTH, the expression of NF-κB and the proportion of cells with NF-κB nuclear translocation decreased (Fig. 5c, d). Besides, a previous study has shown that the activation of NF-κB can regulate the expression of CTSK [21]. To further investigated the relationship between CTH and bone resorption, the expression of CTH in HaCaT cells was knocked down. And after that, the expressions of CTSK and MMP9 were reduced (Fig. 5e). All in all, the results showed that the mechanism of invasion in OKC and AM might be attributed to the activation of NF-κB via the high expression of CTH.

K. Suzuki et al. has reported that hydrogen sulfide plays an important role in the regulation of ferroptosis [10]. Moreover, we found with knockdown of CTH, the expression of nitrogen fixation 1 homolog (NFS-1) was decreased (Fig. 5f). As previous studies have shown, NFS-1, which can be activated by hydrogen sulfide, could help with biogenesis of intracellular Fe-S cluster in order to consume iron ion in the free state [22, 23]. In our research, the expression of NFS-1 was increased in the OKC and AM (Fig. 5g), and there was a positive correlation between the expression of CTH and NFS-1 (Fig. 5h, i). The increase of NFS-1 regulated by CTH might be an in-depth reason for the tolerance of ferroptosis in OKC and AM. In conclusion, we demonstrated that the production of hydrogen sulfide catalyzed by CTH was the main reason for the tolerance of ferroptosis and osteoclast ability in OKC and AM.

## Discussion

Most of the current studies on odontogenic cysts/tumor focus on elucidating the effects of genetic alterations. However, most of the pathophysiological activities in cells occur at the level of metabolites [24]. It has long been a mystery whether metabolites are disordered in odontogenic cysts/tumor and what role the disordered metabolites play. To explore these issues, we initiatively performed the first high-throughput targeted metabolomics analysis in odontogenic cysts/tumor such as OC, OKC, and AM. In our study, a total of 697 metabolites were identified in 37 collected samples. Then, through unsupervised analysis of high-throughput targeted metabolite data, we found different metabolic phenotype stratification of OC, OKC, and AM. The metabolite abundance of the OKC and AM groups can be characterized as “nucleotide/carbohydrate enrichment” and “amino acid enrichment” based on the enrichment of relevant metabolites. Compared with OC, OKC and AM are more bone destructive and exhibit different metabolic patterns, implying that metabolic disorders may be related to their bone destructive capacity. Compared with OC, the enhanced Citrate cycle (TCA cycle) and Nicotinate and nicotinamide metabolism in OKC may be related to the unique lesion pattern. A vigorous TCA cycle promotes chromatin modification, DNA methylation, hypoxia response and the production of signaling molecules for immune function, leading to a high oxidative homeostasis and a high stress state [25]. Vigorous nicotinamide metabolism can effectively inhibit the action of inflammatory molecules and inhibit the process of cell senescence [26]. Notably, in the comparison of the top 50 metabolites in relative abundance, cysteine metabolism was highly correlated with destructive activities in odontogenic cysts/tumor, indicating that the alteration of metabolic flow may be closely related to the progression of the disease. A lot of literature suggests a reciprocal crosstalk between inflammation and metabolism [27, 28]. Our study once again supports this view. As a characteristic metabolite of AM, UDP-glucose accelerates SNAI1 decay and impairs cancer metastasis [29]. On the other hand, from the metabolomic data, higher expression of L-Cysteine, and L-Cystathionine was observed in AM, which further suggested the role of metabolic pathway in OC, OKC and AM, and further implied that cysteine and methionine metabolism pathway determined jaw destructive activities. In consistent of these results, our study also confirmed the relationship between metabolism and bone destruction. Inflammation is one of the key factors affecting metabolism (He et al. 2020), so we categorized IOC independently for the analysis, and the independent metabolic profile confirmed the effect of inflammation. Moreover, based on our present research, the content of intratissue cysteine may be used as a strong predictor of high cell proliferation rate and local invasion in AM. Therefore, the content of intratissue cysteine can be a biomarker of AM, which is helpful to assist in diagnosis AM. WGCNA was performed on the metabolites to find the characteristic metabolite modules that could represent each disease, and then the differentially expressed metabolites were found in each module. In combination with TRAP assay, cysteine, which is closely related to bone destruction, was identified as the target for subsequent research. In order to analyze the relationship between metabolites and biological behavior of odontogenic cysts/tumor more comprehensively, we chose to perform WGCNA on all metabolites. This method can strengthen the strong association and weaken the weak association, and reject the artificial errors [30].

In vitro, we demonstrated that CTH-induced dysregulation of cysteine metabolism promoted the proliferation and ferroptosis tolerance of HaCaT cells, leading to enhanced destructive activities. CTSK and MMP9 can be used as key indicators of destructive activities [31, 32]. In this study, bone destruction activity was indicated by TRAP assay, expression of CTSK and MMP9. CTH and cysteine levels were strongly correlated with all the above indicators, which further confirmed their roles in bone resorption. On the other hand, we observed that cysteine promoted nuclear localization of NF-κB, and then demonstrated the significance of hydrogen sulfide produced by CTH in OKC and AM. Hydrogen sulfide is an important intracellular mammalian gaseous messenger molecule and the activation of NF-κB is also regulated by the hydrogen sulfide (Paul and Snyder 2012). NF-κB is a transcription factor associated with destructive activities, of which high expression was often thought to aggravate bone destruction (Ilchovska and Barrow 2021). Usually, nuclear localization of the p65 subunit is used as a sign of activation the NF-κB pathway (Baldwin 1996). Hydrogen sulfide and its producer CTH are important in the activation of NF-κB and the expression of NSF-1, which lead to local bone destruction and ferroptosis tolerance of OKC and AM, respectively.

The high-throughput targeted metabolomics we employed offers heightened sensitivity and precision compared to conventional non-targeted metabolomics. In contrast to targeted metabolomics, our approach enables comprehensive and systematic detection of a vast array of metabolites. However, it also harbors certain limitations. During the annotation of metabolites in high-throughput targeted metabolomics data, unidentified metabolites remain undiscovered, limiting the annotation process to known metabolites only. Our experimental approach lacked integration with proteomics, thus failing to reflect differences in the activity of upstream and downstream enzymes associated with these metabolites. Simultaneously, the abundance of intricate data holds promise for uncovering non-invasive biomarkers for the diagnosis of odontogenic cysts/tumor in the future. In addition, the effect of nucleotide metabolic pathway on odontogenic cysts/tumor is still unclear, which warrants further investigation. On the other hand, it is necessary to further explore the specific mechanisms by which CTH and hydrogen sulfide regulate NFS-1 expression and Fe-S cluster formation.

In conclusion, this study delineated the characteristics of odontogenic cysts/tumors from a new perspective based on metabolic profiles in different odontogenic cysts/tumor through high-throughput targeted metabolomics. Furthermore, we explored and verified the key metabolic pathways affecting destructive activities. The key role of cysteine-regulated ferroptosis tolerance in jaw destruction was finally confirmed. This will bring new strategy for the diagnosis and targeted treatments of odontogenic cysts/tumor. In addition, our work may provide new possibilities for the application of cystic fluid-based liquid biopsy.

### Supplementary Information

Supplementary file1 (TIF 2222 KB)

Supplementary file2 (TIF 3915 KB)

Supplementary file3 (TIF 4054 KB)

Supplementary file4 (TIF 3928 KB)

Supplementary file5 (TIF 1484 KB)

Supplementary file6 (DOCX 64 KB)

## Data Availability

The authors declare that all data and materials as well as software application or custom code support their published claims and comply with field standards.
