# Supplementary figures and images for: L-cysteine contributes to destructive activities of odontogenic cysts/tumor
Source: Discov Oncol. 2024 Apr 8;15:109. doi: 10.1007/s12672-024-00959-5 (PMC11001836; doi:10.1007/s12672-024-00959-5)

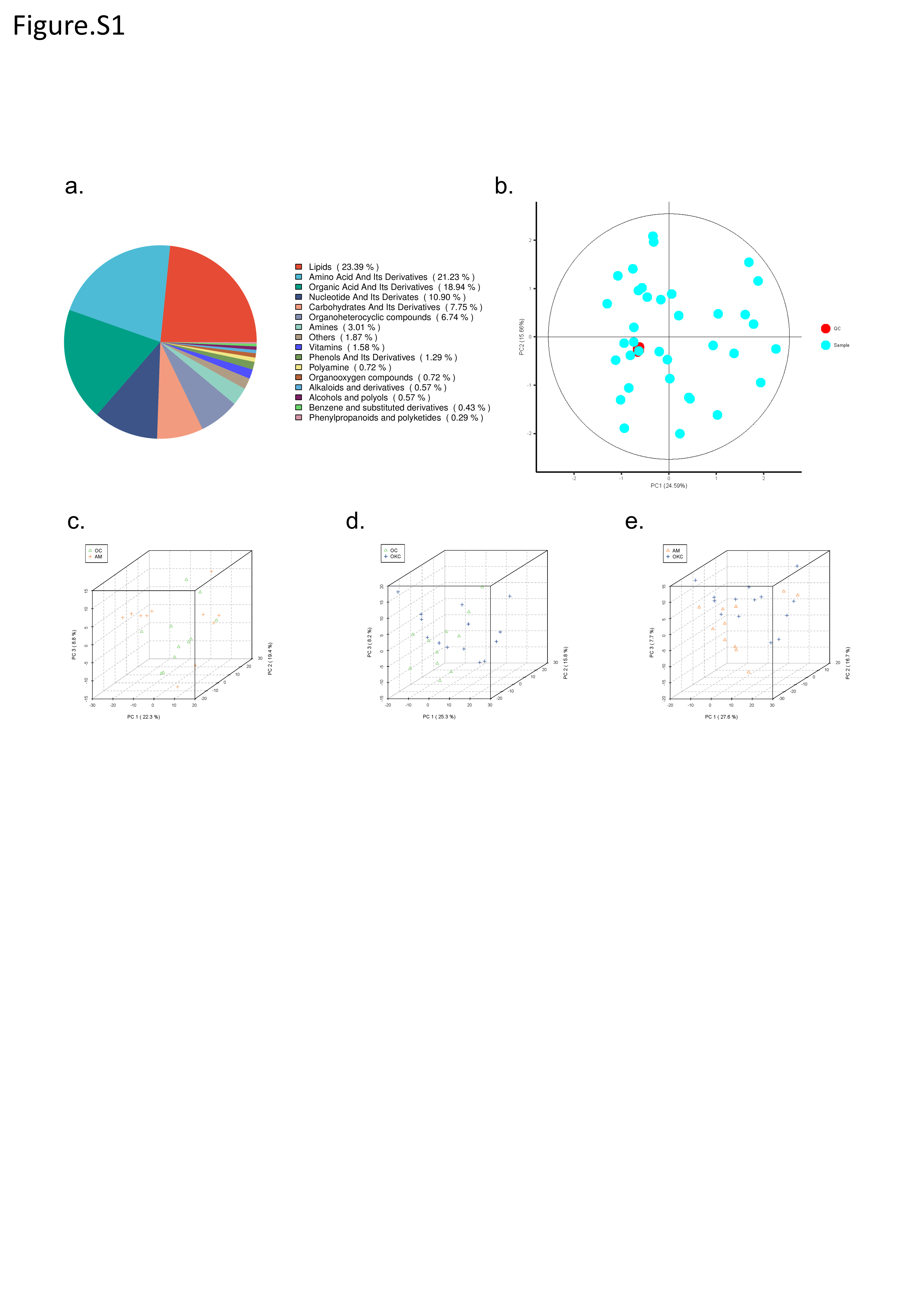

Supplement: Supplementary file 1 — Supplementary file1 (TIF 2222 KB) [file 12672_2024_959_MOESM1_ESM.tif]

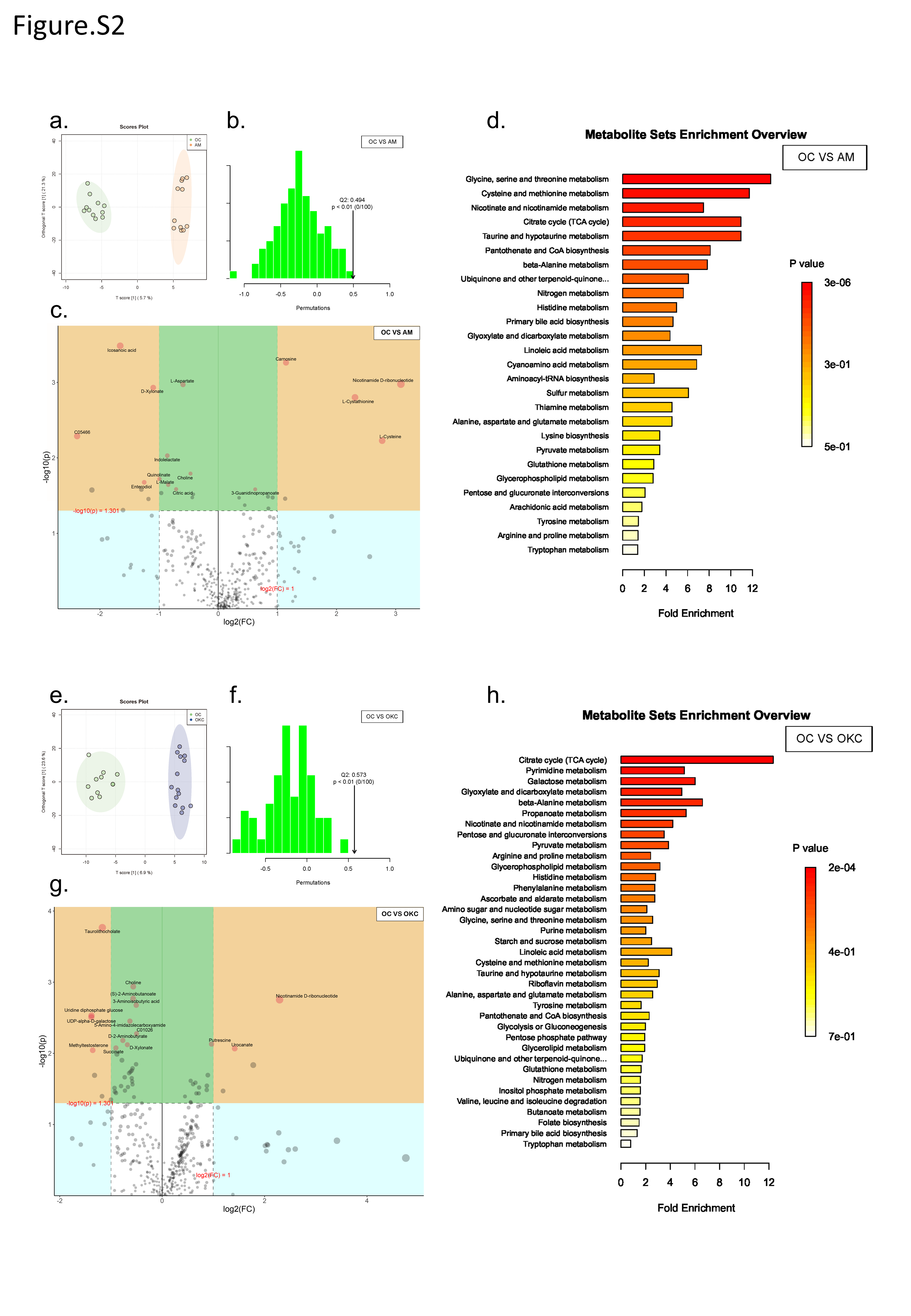

Supplement: Supplementary file 2 — Supplementary file2 (TIF 3915 KB) [file 12672_2024_959_MOESM2_ESM.tif]

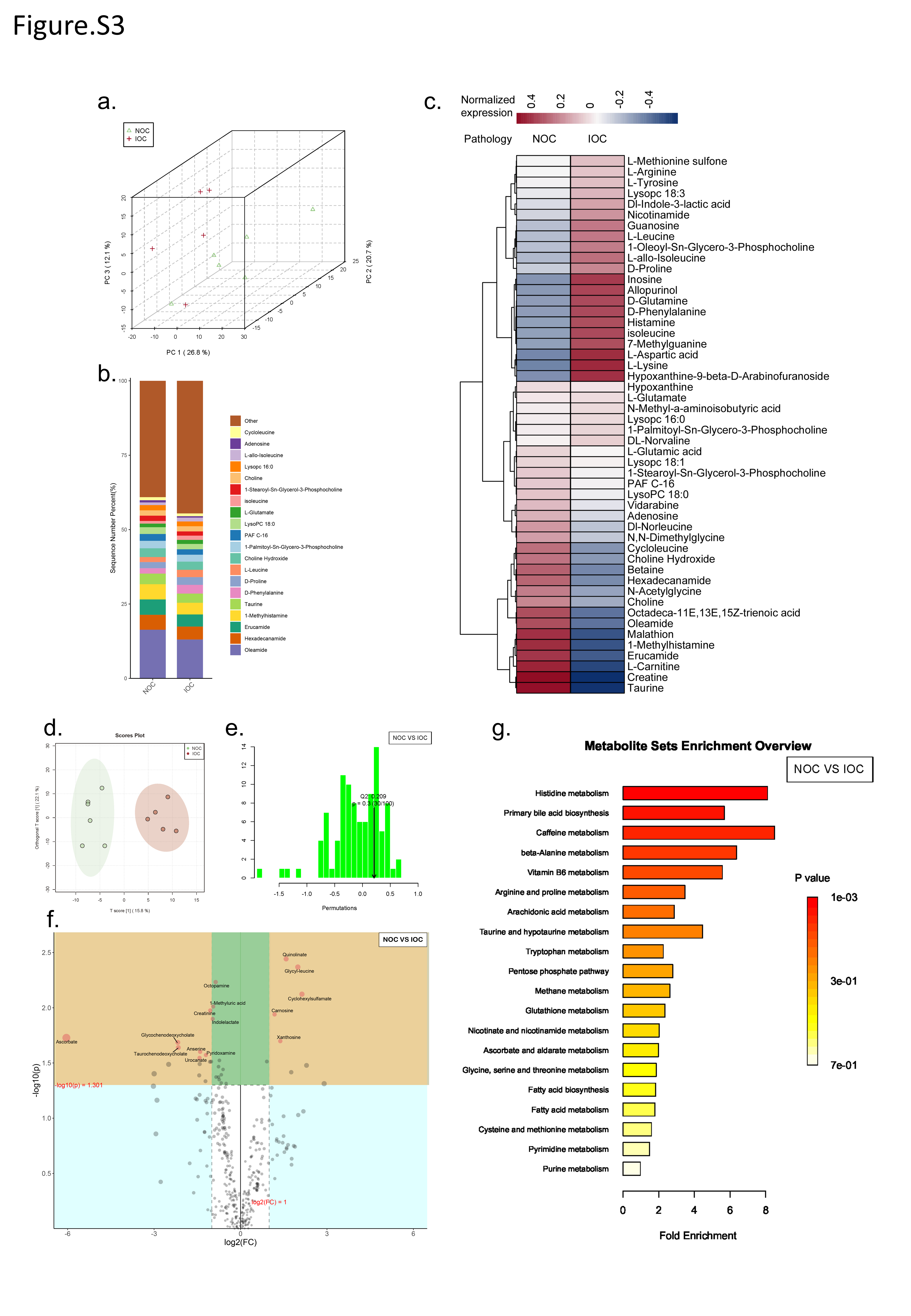

Supplement: Supplementary file 3 — Supplementary file3 (TIF 4054 KB) [file 12672_2024_959_MOESM3_ESM.tif]

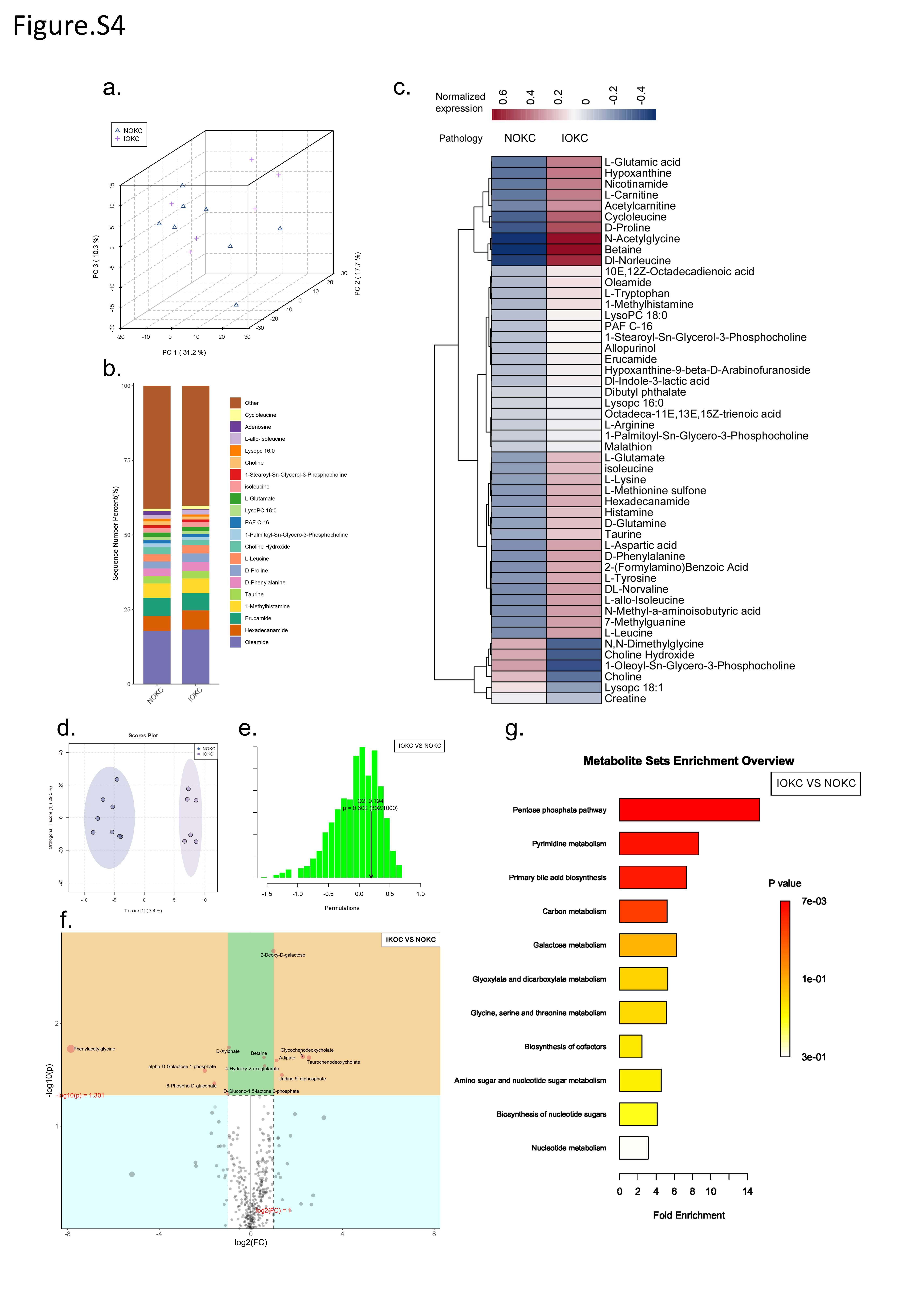

Supplement: Supplementary file 4 — Supplementary file4 (TIF 3928 KB) [file 12672_2024_959_MOESM4_ESM.tif]

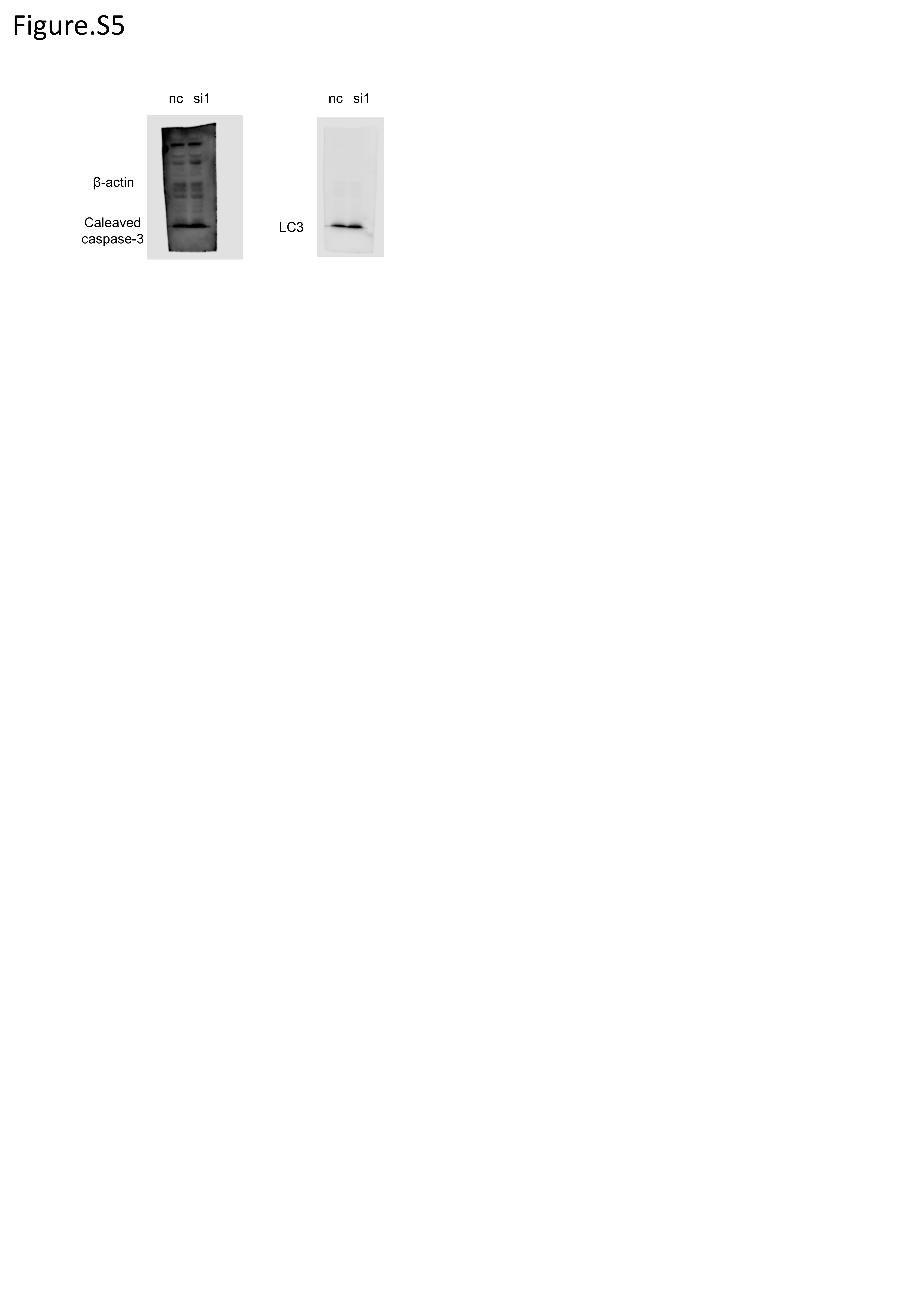

Supplement: Supplementary file 5 — Supplementary file5 (TIF 1484 KB) [file 12672_2024_959_MOESM5_ESM.tif]
