# Supplementary material for: L-cysteine contributes to destructive activities of odontogenic cysts/tumor
Source: Discov Oncol. 2024 Apr 8;15:109. doi: 10.1007/s12672-024-00959-5 (PMC11001836; doi:10.1007/s12672-024-00959-5)
Supplement: Supplementary file 6 — Supplementary file6 (DOCX 64 KB) [file 12672_2024_959_MOESM6_ESM.docx]

**Supplementary Table**

Supplementary Table.1 materials

| Antibody | Brand | Cat.# | IHC | WB |
| --- | --- | --- | --- | --- |
| Col1a1 | Cell Signaling Technology | 72026s | 1:250 | - |
| CTSK | Abcepta | ap7981c-ev | 1:300 | - |
| MMP9 | Proteintech | 10375-2-ap | 1:250 | - |
| CTH | Proteintech | 12217-1-ap | 1:300 | 1:2000 |
| SLC7A11 | Proteintech | 26864-1-ap | 1:250 | - |
| NF-κB | Proteintech | 10745-1-ap | 1:250 | - |
| NFS-1 | Proteintech | 15370-1-ap | 1:250 | - |
| Caleaced Caspase-3 | Proteintech | 25128-1-AP | - | 1:1500 |
| LC3 | Proteintech | 81004-1-RR | - | 1:2500 |
| β-actin | Bioprimacy | PMK058 | - | 1:10000 |
| Goat anti-Rabbit IgG | Bioprimacy | PMK-014-090 | - | 1:10000 |
| Goat anti-Mouse IgG | Bioprimacy | PMK-014-091 | - | 1:10000 |

Supplementary Table.2 Clinical characteristics of the tissue samples involved in metabolomics

| Number | Gender | Age | Smoking history | Alcohol history | Systemic disease history | Drug allergy history | Recurrence history | Location | Pathological diagnosis | Group | |
| --- | --- | --- | --- | --- | --- | --- | --- | --- | --- | --- | --- |
| 1 | Male | 51 | Yes | No | No | No | Yes | Left mandible | Ameloblastoma | AM | |
| 2 | Male | 61 | Yes | Yes | Yes | Yes | No | Right mandible | Ameloblastoma |  |  |
| 3 | Female | 11 | No | No | No | No | No | Left mandible | Ameloblastoma |  |  |
| 4 | Male | 29 | No | No | No | No | No | Mandible | Solid ameloblastoma |  |  |
| 5 | Male | 38 | Yes | Yes | Yes | No | No | Mandible | Ameloblastoma |  |  |
| 6 | Female | 21 | No | No | No | No | No | Left mandible | Ameloblastoma |  |  |
| 7 | Female | 58 | No | No | Yes | Yes | Yes | Left mandible | Ameloblastoma |  |  |
| 8 | Female | 59 | No | No | No | No | Yes | Right mandible | Ameloblastoma |  |  |
| 9 | Female | 34 | No | No | No | No | Yes | Left mandible | Ameloblastoma. |  |  |
| 10 | Female | 12 | No | No | No | No | No | Right mandible | Solid ameloblastoma |  |  |
| 11 | Male | 11 | No | No | No | No | No | Right mandible | Solid ameloblastoma |  |  |
| 12 | Male | 15 | No | No | No | No | No | Midmandible | Odontogenic keratocyst | NOKC | OKC |
| 13 | Male | 17 | No | No | No | No | No | Left and right mandible | Odontogenic keratocyst |  |  |
| 14 | Female | 24 | No | No | Yes | No | No | Left and right maxilla and left maxilla | Mutiple odontogenic keratocyst |  |  |
| 15 | Male | 14 | No | No | No | No | No | Right mandible | Odontogenic keratocyst |  |  |
| 16 | Male | 65 | No | No | No | No | No | Right mandible | Odontogenic keratocyst |  |  |
| 17 | Male | 11 | No | No | No | No | No | Left mandible | Odontogenic keratocyst |  |  |
| 18 | Female | 35 | No | No | No | No | No | Left mandible | Odontogenic keratocyst |  |  |
| 19 | Female | 14 | No | No | No | No | No | Right and left mandible | Odontogenic keratocyst |  |  |
| 20 | Male | 50 | Yes | Yes | No | No | Yes | Right mandible | Odontogenic keratocyst with inflammatory | IOKC |  |
| 21 | Male | 21 | No | No | No | No | No | Right mandible | Odontogenic keratocyst with inflammatory |  |  |
| 22 | Male | 66 | Yes | No | No | Yes | No | Right mandible | Odontogenic keratocyst with inflammatory |  |  |
| 23 | Female | 23 | No | No | No | No | No | Right maxilla and mandible | Odontogenic keratocyst with inflammatory |  |  |
| 24 | Male | 17 | No | No | No | No | No | Left mandible | Odontogenic keratocyst with inflammatory |  |  |
| 25 | Female | 31 | No | No | No | No | No | Right mandible | Odontogenic keratocyst with inflammatory |  |  |
| 26 | Male | 50 | No | Yes | No | Yes | No | Right and left mandible | Dentigerous cyst | NOC | OC |
| 27 | Male | 51 | Yes | Yes | No | No | No | Right mandible | Dentigerous cyst |  |  |
| 28 | Male | 30 | No | No | No | No | No | Left maxilla | Dentigerous cyst |  |  |
| 29 | Male | 50 | No | Yes | No | Yes | No | Left maxilla | Developmental odontogenic cyst |  |  |
| 30 | Female | 59 | No | No | No | No | No | Right mandible | Developmental odontogenic cyst |  |  |
| 31 | Female | 48 | Yes | No | No | No | No | Right maxilla | Developmental odontogenic cyst |  |  |
| 33 | Male | 49 | No | No | No | No | No | Right maxilla | Radicular cyst | IOC |  |
| 34 | Male | 11 | No | No | No | No | No | Right mandible | Radicular cyst |  |  |
| 35 | Male | 55 | Yes | No | Yes | No | No | Left maxilla | Radicular cyst |  |  |
| 36 | Female | 38 | No | No | No | No | No | Left mandible | Radicular cyst |  |  |
| 37 | Female | 37 | No | No | No | No | No | Midmandible | Odontogenic cyst with inflammatory |  |  |

Supplementary Table.3 Metabolites of AM VS OC

| ID of kegg | Description | Which-Max | | log2(FC) | Raw pval |
| --- | --- | --- | --- | --- | --- |
| C00097 | L-Cysteine | | AM | 2.7714 | 0.0059232 |
| C00318 | L-Carnitine | | AM | 0.35252 | 0.033808 |
| C00386 | Carnosine | | AM | 1.1457 | 0.0005474 |
| C00455 | Nicotinamide D-ribonucleotide | | AM | 3.0878 | 0.0010551 |
| C00606 | L-Cysteinesulfinic acid | | AM | 0.91972 | 0.033501 |
| C01817 | L-Homocystine | | AM | 0.84799 | 0.046807 |
| C02291 | L-Cystathionine | | AM | 2.3121 | 0.0015808 |
| C03065 | 3-Guanidinopropanoate | | AM | 0.6248 | 0.026127 |
| C04227 | Octopamine | | AM | 0.44819 | 0.042369 |
| C05824 | S-Sulfo-L-cysteine | | AM | 1.1314 | 0.034728 |
| C06501 | Phosphotyrosine | | AM | 0.8409 | 0.032712 |
| C00049 | L-Aspartate | | OC | -0.59566 | 0.0010694 |
| C00114 | Choline | | OC | -0.46887 | 0.016167 |
| C00149 | L-Malate | | OC | -0.84555 | 0.022773 |
| C00158 | Citric acid | | OC | -0.71203 | 0.025991 |
| C00502 | D-Xylonate | | OC | -1.0994 | 0.0011734 |
| C00719 | Betaine | | OC | -0.43853 | 0.033495 |
| C01026 | N,N-Dimethylglycine | | OC | -0.4225 | 0.030391 |
| C01921 | Glycocholic acid | | OC | -2.136 | 0.026656 |
| C02043 | Indolelactate | | OC | -0.86092 | 0.0093443 |
| C02483 | gamma-Tocopherol | | OC | -1.2993 | 0.026294 |
| C03672 | 3-(4-Hydroxyphenyl)lactate | | OC | -0.96258 | 0.029271 |
| C03722 | Quinolinate | | OC | -0.99339 | 0.019057 |
| C04717 | 13(S)-HPODE | | OC | -1.6098 | 0.049306 |
| C04742 | 15(S)-HETE | | OC | -1.1796 | 0.035071 |
| C05466 | Glycochenodeoxycholic acid | | OC | -2.3857 | 0.0051604 |
| C06425 | Icosanoic acid | | OC | -1.6575 | 0.0003278 |
| C18166 | Enterodiol | | OC | -1.2515 | 0.021085 |

Supplementary Table.4 Pathway of AM VS OC

| ID | Description | Raw p | Fold Enrichment |
| --- | --- | --- | --- |
| hsa00260 | Glycine, serine and threonine metabolism | 1.81E-05 | 13.556 |
| hsa00760 | Nicotinate and nicotinamide metabolism | 0.00048636 | 17.894 |
| hsa01230 | Biosynthesis of amino acids | 0.0027456 | 6.3908 |
| hsa00430 | Taurine and hypotaurine metabolism | 0.0031663 | 22.368 |
| hsa00270 | Cysteine and methionine metabolism | 0.0051464 | 8.1337 |
| hsa00340 | Histidine metabolism | 0.012874 | 11.184 |
| hsa01210 | 2-Oxocarboxylic acid metabolism | 0.017987 | 9.418 |
| hsa00020 | Citrate cycle (TCA cycle) | 0.019855 | 8.9471 |
| hsa00770 | Pantothenate and CoA biosynthesis | 0.019855 | 8.9471 |
| hsa00410 | beta-Alanine metabolism | 0.021801 | 8.521 |
| hsa01200 | Carbon metabolism | 0.032986 | 4.1294 |
| hsa00250 | Alanine, aspartate and glutamate metabolism | 0.037483 | 6.3908 |
| hsa00630 | Glyoxylate and dicarboxylate metabolism | 0.047915 | 5.5919 |
| hsa00591 | Linoleic acid metabolism | 0.054719 | 17.894 |
| hsa00730 | Thiamine metabolism | 0.075808 | 12.782 |
| hsa00120 | Primary bile acid biosynthesis | 0.091271 | 3.89 |
| hsa00970 | Aminoacyl-tRNA biosynthesis | 0.09819 | 3.7279 |
| hsa00220 | Arginine biosynthesis | 0.14618 | 6.3908 |
| hsa01240 | Biosynthesis of cofactors | 0.19223 | 1.9592 |
| hsa00620 | Pyruvate metabolism | 0.22927 | 3.89 |
| hsa00480 | Glutathione metabolism | 0.27208 | 3.1954 |
| hsa00590 | Arachidonic acid metabolism | 0.33594 | 2.4853 |
| hsa01040 | Biosynthesis of unsaturated fatty acids | 0.33594 | 2.4853 |
| hsa00564 | Glycerophospholipid metabolism | 0.33594 | 2.4853 |

Supplementary Table.5 Metabolites of OKC VS OC

| ID of kegg | Description | Which-Max | log2(FC) | raw.pval |
| --- | --- | --- | --- | --- |
| C00003 | Nicotinamide adenine dinucleotide | OKC | 0.64964 | 0.025824 |
| C00013 | Diphosphoric acid | OKC | 1.7789 | 0.014616 |
| C00019 | S-Adenosyl-L-methionine | OKC | 0.66781 | 0.036503 |
| C00134 | Putrescine | OKC | 0.96502 | 0.0073971 |
| C00455 | Nicotinamide D-ribonucleotide | OKC | 2.2933 | 0.0017661 |
| C00785 | Urocanate | OKC | 1.417 | 0.0085557 |
| C00978 | N-Acetylserotonin | OKC | 0.89224 | 0.020213 |
| C00986 | 1,3-Diaminopropane | OKC | 0.71486 | 0.036473 |
| C01013 | 3-Hydroxypropanoate | OKC | 0.44552 | 0.040284 |
| C01198 | 3-(2-Hydroxyphenyl)propanoate | OKC | 0.55862 | 0.049389 |
| C02642 | 3-Ureidopropionate | OKC | 0.55586 | 0.048233 |
| C02946 | 4-Acetamidobutanoate | OKC | 0.60341 | 0.027139 |
| C05580 | 3,4-Dihydroxymandelate | OKC | 0.87278 | 0.028529 |
| C05824 | S-Sulfo-L-cysteine | OKC | 0.72217 | 0.03016 |
| C16512 | Palmitoylethanolamide | OKC | 0.8706 | 0.032674 |
| C19615 | Hexadecanedioate | OKC | 0.55692 | 0.024282 |
| C19806 | Propane-1,2,3-tricarboxylate | OKC | 1.1896 | 0.033751 |
| C00029 | Uridine diphosphate glucose | OC | -1.3796 | 0.0029357 |
| C00031 | D-Glucose | OC | -0.90215 | 0.036062 |
| C00042 | Succinate | OC | -0.90267 | 0.0083869 |
| C00052 | UDP-alpha-D-galactose | OC | -1.3825 | 0.0031081 |
| C00114 | Choline | OC | -0.56469 | 0.0011539 |
| C00137 | myo-Inositol | OC | -0.92962 | 0.030698 |
| C00149 | L-Malate | OC | -0.59398 | 0.022292 |
| C00153 | Nicotinic acid amide | OC | -0.57775 | 0.016244 |
| C00158 | Citrate | OC | -0.61854 | 0.018142 |
| C00242 | Guanine | OC | -0.5922 | 0.017408 |
| C00245 | Taurine | OC | -0.50908 | 0.014216 |
| C00256 | D-Lactate | OC | -0.64546 | 0.018964 |
| C00294 | Inosine | OC | -0.57878 | 0.027325 |
| C00311 | Isocitrate | OC | -0.73161 | 0.021671 |
| C00451 | D-threo-Isocitric acid | OC | -0.72653 | 0.033354 |
| C00474 | Ribitol | OC | -0.78537 | 0.012484 |
| C00502 | D-Xylonate | OC | -0.67929 | 0.007561 |
| C00568 | 4-Aminobenzoate | OC | -0.58581 | 0.024734 |
| C01026 | N,N-Dimethylglycine | OC | -0.50011 | 0.0052862 |
| C01103 | Orotidine 5'-phosphate | OC | -0.5498 | 0.041136 |
| C01595 | Linoleate | OC | -0.66735 | 0.029706 |
| C02170 | Methylmalonate | OC | -0.88305 | 0.010261 |
| C02261 | D-2-Aminobutyrate | OC | -0.76746 | 0.0065838 |
| C02356 | (S)-2-Aminobutanoate | OC | -0.56651 | 0.0016917 |
| C02483 | gamma-Tocopherol | OC | -1.174 | 0.040062 |
| C02592 | Taurolithocholate | OC | -1.1678 | 0.00017097 |
| C02824 | Cyclohexylsulfamate | OC | -1.3198 | 0.020347 |
| C02835 | Imidazole-4-acetate | OC | -0.80235 | 0.0288 |
| C04051 | 5-Amino-4-imidazolecarboxyamide | OC | -0.62949 | 0.0035275 |
| C04230 | 1-Acyl-sn-glycero-3-phosphocholine | OC | -0.9991 | 0.044633 |
| C05145 | 3-Aminoisobutyric acid | OC | -0.50618 | 0.0021032 |
| C06153 | scyllo-Inositol | OC | -0.92289 | 0.034555 |
| C07198 | Methyltestosterone | OC | -1.356 | 0.0090422 |

Supplementary Table.6 Pathway of OKC VS OC

| ID | Description | Raw p | Fold Enrichment |
| --- | --- | --- | --- |
| hsa00020 | Citrate cycle (TCA cycle) | 0.00069766 | 9.2182 |
| hsa00052 | Galactose metabolism | 0.0022693 | 6.8283 |
| hsa00760 | Nicotinate and nicotinamide metabolism | 0.0035473 | 9.2182 |
| hsa00410 | beta-Alanine metabolism | 0.0094868 | 6.5844 |
| hsa01200 | Carbon metabolism | 0.011446 | 3.5455 |
| hsa00053 | Ascorbate and aldarate metabolism | 0.014945 | 10.242 |
| hsa00630 | Glyoxylate and dicarboxylate metabolism | 0.030082 | 4.321 |
| hsa00524 | Neomycin, kanamycin and gentamicin biosynthesis | 0.042936 | 23.045 |
| hsa00340 | Histidine metabolism | 0.045318 | 5.7614 |
| hsa00330 | Arginine and proline metabolism | 0.046853 | 3.6388 |
| hsa00500 | Starch and sucrose metabolism | 0.056248 | 5.1212 |
| hsa01250 | Biosynthesis of nucleotide sugars | 0.056665 | 3.3725 |
| hsa01210 | 2-Oxocarboxylic acid metabolism | 0.062028 | 4.8517 |
| hsa00640 | Propanoate metabolism | 0.080496 | 4.1901 |
| hsa00620 | Pyruvate metabolism | 0.086995 | 4.0079 |
| hsa00591 | Linoleic acid metabolism | 0.10401 | 9.2182 |
| hsa01230 | Biosynthesis of amino acids | 0.1185 | 2.4692 |
| hsa00250 | Alanine, aspartate and glutamate metabolism | 0.12163 | 3.2922 |
| hsa00260 | Glycine, serine and threonine metabolism | 0.15906 | 2.7934 |
| hsa00270 | Cysteine and methionine metabolism | 0.15906 | 2.7934 |
| hsa00430 | Taurine and hypotaurine metabolism | 0.16129 | 5.7614 |
| hsa00230 | Purine metabolism | 0.16406 | 2.1273 |
| hsa00564 | Glycerophospholipid metabolism | 0.18247 | 2.5606 |
| hsa00520 | Amino sugar and nucleotide sugar metabolism | 0.1904 | 2.4914 |
| hsa00240 | Pyrimidine metabolism | 0.20639 | 2.3636 |
| hsa00650 | Butanoate metabolism | 0.28148 | 3.0727 |
| hsa00040 | Pentose and glucuronate interconversions | 0.32772 | 2.5606 |
| hsa01232 | Nucleotide metabolism | 0.32873 | 1.7071 |
| hsa01240 | Biosynthesis of cofactors | 0.3458 | 1.3457 |
| hsa00770 | Pantothenate and CoA biosynthesis | 0.35693 | 2.3045 |
| hsa00480 | Glutathione metabolism | 0.46193 | 1.6461 |
| hsa00562 | Inositol phosphate metabolism | 0.48546 | 1.5364 |
| hsa01040 | Biosynthesis of unsaturated fatty acids | 0.55022 | 1.2803 |
| hsa00280 | Valine, leucine and isoleucine degradation | 0.58892 | 1.1523 |
| hsa00380 | Tryptophan metabolism | 0.59808 | 1.1242 |
| hsa00350 | Tyrosine metabolism | 0.60705 | 1.0974 |
| hsa00120 | Primary bile acid biosynthesis | 0.64099 | 1.002 |

Supplementary Table.7 Metabolites of IOC VS DOC

| ID of kegg | Description | Which-MNOCx | log2(FC) | raw.pval |
| --- | --- | --- | --- | --- |
| C06424 | Tetradecanoic acid | IOC | 0.9136 | 0.030666 |
| C00386 | Carnosine | IOC | 1.1815 | 0.011475 |
| C01762 | Xanthosine | IOC | 1.38 | 0.020049 |
| C03722 | Quinolinate | IOC | 1.5825 | 0.0036114 |
| C00584 | Prostaglandin E2 | IOC | 1.7598 | 0.038332 |
| C02155 | Glycyl-leucine | IOC | 1.9888 | 0.0042794 |
| C02824 | Cyclohexylsulfamate | IOC | 2.1315 | 0.0075309 |
| C00427 | Prostaglandin H2 | IOC | 2.2906 | 0.03326 |
| C02989 | L-Methionine S-oxide | IOC | 2.9028 | 0.04868 |
| C00072 | Ascorbate | NOC | -6.0438 | 0.018705 |
| C05565 | Hydantoin-5-propionate | NOC | -2.9972 | 0.039577 |
| C01921 | Glycocholate | NOC | -2.495 | 0.03254 |
| C05466 | Glycochenodeoxycholate | NOC | -2.176 | 0.020602 |
| C05465 | Taurochenodeoxycholate | NOC | -2.159 | 0.02293 |
| C00519 | Hypotaurine | NOC | -1.4336 | 0.040963 |
| C00785 | Urocanate | NOC | -1.4224 | 0.028453 |
| C01104 | Trimethylamine N-oxide | NOC | -1.4171 | 0.032299 |
| C01262 | Anserine | NOC | -1.4011 | 0.025051 |
| C19806 | Propane-1,2,3-tricarboxylate | NOC | -1.2155 | 0.046256 |
| C00534 | Pyridoxamine | NOC | -1.2019 | 0.026799 |
| C00978 | N-Acetylserotonin | NOC | -1.0613 | 0.030845 |
| C00791 | Creatinine | NOC | -1.0502 | 0.010584 |
| C00437 | N-Acetylornithine | NOC | -1.0187 | 0.03071 |
| C02043 | Indolelactate | NOC | -0.96666 | 0.012627 |
| C16359 | 1-Methyluric acid | NOC | -0.94878 | 0.0097459 |
| C04227 | Octopamine | NOC | -0.86186 | 0.0058513 |
| C00314 | Pyridoxine | NOC | -0.84766 | 0.042405 |
| C00198 | D-Glucono-1,5-lactone | NOC | -0.76332 | 0.042405 |
| C00364 | Thymidine 5'-phosphate | NOC | -0.76263 | 0.034147 |
| C00715 | Pterin | NOC | -0.73995 | 0.044779 |
| C05672 | 2-Amino-3-phosphonopropanoate | NOC | -0.73275 | 0.02997 |
| C00517 | Hexadecanal | NOC | -0.66844 | 0.047937 |
| C14088 | 3-Methylsalicylate | NOC | -0.66647 | 0.040427 |
| C00300 | Creatine | NOC | -0.6057 | 0.036191 |
| C08278 | Suberic acid | NOC | -0.49946 | 0.048398 |

Supplementary Table.8 Pathway of IOC VS DOC

| ID | Description | Raw p | Fold Enrichment |
| --- | --- | --- | --- |
| hsa00052 | Galactose metabolism | 0.00090303 | 8.6667 |
| hsa01250 | Biosynthesis of nucleotide sugars | 0.0044248 | 5.7073 |
| hsa00120 | Primary bile acid biosynthesis | 0.0067257 | 5.087 |
| hsa00430 | Taurine and hypotaurine metabolism | 0.0073886 | 14.625 |
| hsa00053 | Ascorbate and aldarate metabolism | 0.0094001 | 13 |
| hsa00520 | Amino sugar and nucleotide sugar metabolism | 0.023444 | 4.7432 |
| hsa00524 | Neomycin, kanamycin and gentamicin biosynthesis | 0.033907 | 29.25 |
| hsa00500 | Starch and sucrose metabolism | 0.036352 | 6.5 |
| hsa00640 | Propanoate metabolism | 0.052643 | 5.3182 |
| hsa00230 | Purine metabolism | 0.096183 | 2.7 |
| hsa00260 | Glycine, serine and threonine metabolism | 0.10738 | 3.5455 |
| hsa00750 | Vitamin B6 metabolism | 0.14409 | 6.5 |
| hsa00650 | Butanoate metabolism | 0.22882 | 3.9 |
| hsa00340 | Histidine metabolism | 0.24214 | 3.6562 |
| hsa00040 | Pentose and glucuronate interconversions | 0.2681 | 3.25 |
| hsa00051 | Fructose and mannose metabolism | 0.29321 | 2.925 |
| hsa00020 | Citrate cycle (TCA cycle) | 0.29321 | 2.925 |
| hsa00620 | Pyruvate metabolism | 0.32933 | 2.5435 |
| hsa00250 | Alanine, aspartate and glutamate metabolism | 0.38562 | 2.0893 |
| hsa00480 | Glutathione metabolism | 0.38562 | 2.0893 |
| hsa00562 | Inositol phosphate metabolism | 0.40684 | 1.95 |
| hsa00270 | Cysteine and methionine metabolism | 0.43735 | 1.7727 |
| hsa00590 | Arachidonic acid metabolism | 0.46635 | 1.625 |
| hsa01040 | Biosynthesis of unsaturated fatty acids | 0.46635 | 1.625 |
| hsa00564 | Glycerophospholipid metabolism | 0.46635 | 1.625 |
| hsa00330 | Arginine and proline metabolism | 0.48488 | 1.5395 |
| hsa00280 | Valine, leucine and isoleucine degradation | 0.50279 | 1.4625 |
| hsa00350 | Tyrosine metabolism | 0.5201 | 1.3929 |
| hsa01232 | Nucleotide metabolism | 0.61241 | 1.0833 |
| hsa01200 | Carbon metabolism | 0.68184 | 0.9 |
| hsa01240 | Biosynthesis of cofactors | 0.91588 | 0.42701 |

Supplementary Table.9 Metabolites of IOKC VS DOKC

| ID of kegg | Description | Which-Max | log2(FC) | raw.pval |
| --- | --- | --- | --- | --- |
| C05465 | Taurochenodeoxycholate | IOKC | 2.5343 | 0.021599 |
| C05466 | Glycochenodeoxycholate | IOKC | 2.2642 | 0.02109 |
| C00015 | Uridine 5'-diphosphate | IOKC | 1.3511 | 0.031759 |
| C06104 | Adipate | IOKC | 1.1253 | 0.023 |
| C02781 | 2-Deoxy-D-galactose | IOKC | 0.98271 | 0.0019792 |
| C01127 | 4-Hydroxy-2-oxoglutarate | IOKC | 0.59987 | 0.025979 |
| C00719 | Betaine | IOKC | 0.57389 | 0.021441 |
| C05598 | Phenylacetylglycine | NOKC | -7.8542 | 0.017695 |
| C00446 | alpha-D-Galactose 1-phosphate | NOKC | -2.0156 | 0.028951 |
| C00345 | 6-Phospho-D-gluconate | NOKC | -1.5954 | 0.038318 |
| C01236 | D-Glucono-1,5-lactone 6-phosphate | NOKC | -0.99766 | 0.048787 |
| C00502 | D-Xylonate | NOKC | -0.95875 | 0.017164 |

Supplementary Table.10 Pathway of IOC VS DOC

| ID of kegg | Description | Raw p | Fold Enrichment |
| --- | --- | --- | --- |
| hsa00030 | Pentose phosphate pathway | 0.0067642 | 15.364 |
| hsa00240 | Pyrimidine metabolism | 0.020589 | 8.6667 |
| hsa00120 | Primary bile acid biosynthesis | 0.028143 | 7.3478 |
| hsa01200 | Carbon metabolism | 0.053332 | 5.2 |
| hsa00052 | Galactose metabolism | 0.14924 | 6.2593 |
| hsa00630 | Glyoxylate and dicarboxylate metabolism | 0.17459 | 5.2812 |
| hsa00260 | Glycine, serine and threonine metabolism | 0.17958 | 5.1212 |
| hsa01240 | Biosynthesis of cofactors | 0.1911 | 2.4672 |
| hsa00520 | Amino sugar and nucleotide sugar metabolism | 0.19927 | 4.5676 |
| hsa01250 | Biosynthesis of nucleotide sugars | 0.21854 | 4.122 |
| hsa01232 | Nucleotide metabolism | 0.27835 | 3.1296 |

Supplementary Table.11 Metabolites of AM VS OKC

| ID of kegg | Description | Which-Max | log2(FC) | raw.pval |
| --- | --- | --- | --- | --- |
| C00097 | L-Cysteine | AM | -2.2842 | 0.026546 |
| C00108 | Anthranilic acid | AM | -0.65138 | 0.025628 |
| C00186 | L-Lactate | AM | -0.99966 | 0.017956 |
| C00256 | D-Lactate | AM | -1.115 | 0.013177 |
| C00262 | Hypoxanthine | AM | -0.47777 | 0.019813 |
| C00318 | L-Carnitine | AM | -0.52807 | 0.0045516 |
| C00352 | D-Glucosamine 6-phosphate | AM | -0.84013 | 0.012272 |
| C00386 | Carnosine | AM | -0.89293 | 0.00032439 |
| C00606 | 3-Sulfinoalanine | AM | -1.0057 | 0.0030855 |
| C01179 | 3-(4-Hydroxyphenyl)pyruvate | AM | -0.7268 | 0.010783 |
| C02261 | D-2-Aminobutyrate | AM | -1.1678 | 0.025409 |
| C02291 | L-Cystathionine | AM | -1.8232 | 0.011155 |
| C02356 | (S)-2-Aminobutanoate | AM | -0.63181 | 0.027823 |
| C04227 | Octopamine | AM | -0.46428 | 0.022367 |
| C04256 | N-Acetyl-D-glucosamine 1-phosphate | AM | -1.9674 | 0.0089975 |
| C05570 | Ergothioneine | AM | -0.80926 | 0.035259 |
| C07198 | Methyltestosterone | AM | -0.95124 | 0.024735 |
| C00134 | Putrescine | OKC | 0.68613 | 0.030947 |
| C00295 | Orotate | OKC | 0.44391 | 0.049772 |
| C00388 | Histamine | OKC | 0.87765 | 0.039352 |
| C00785 | Urocanic acid | OKC | 1.4979 | 0.0056462 |
| C00788 | L-Adrenaline | OKC | 0.82412 | 0.038353 |
| C00978 | N-Acetylserotonin | OKC | 0.80979 | 0.039508 |
| C02946 | 4-Acetamidobutanoate | OKC | 0.57275 | 0.032976 |
| C04742 | 15(S)-HETE | OKC | 1.8032 | 0.00047493 |
| C06425 | Icosanoic acid | OKC | 0.99152 | 0.0022722 |
| C16512 | Palmitoylethanolamide | OKC | 1.0621 | 0.0063768 |
| C18166 | Enterodiol | OKC | 1.8166 | 7.75E-05 |
| C19615 | Hexadecanedioic acid | OKC | 0.71118 | 0.004941 |

Supplementary Table.12 Pathway of AM VS OKC

| ID | Description | p value | Fold Enrichment |
| --- | --- | --- | --- |
| hsa00270 | Cysteine and methionine metabolism | 0.00069766 | 9.2182 |
| hsa00340 | Histidine metabolism | 0.00097748 | 14.259 |
| hsa00430 | Taurine and hypotaurine metabolism | 0.0043886 | 19.012 |
| hsa00620 | Pyruvate metabolism | 0.035235 | 6.613 |
| hsa00480 | Glutathione metabolism | 0.050622 | 5.4321 |
| hsa00400 | Phenylalanine, tyrosine and tryptophan biosynthesis | 0.051619 | 19.012 |
| hsa00260 | Glycine, serine and threonine metabolism | 0.068003 | 4.6091 |
| hsa00330 | Arginine and proline metabolism | 0.087085 | 4.0026 |
| hsa00730 | Thiamine metabolism | 0.08866 | 10.864 |
| hsa00380 | Tryptophan metabolism | 0.09924 | 3.7098 |
| hsa00350 | Tyrosine metabolism | 0.1034 | 3.6214 |
| hsa01230 | Biosynthesis of amino acids | 0.166 | 2.7161 |
| hsa00130 | Ubiquinone and other terpenoid-quinone biosynthesis | 0.21306 | 4.225 |
| hsa00770 | Pantothenate and CoA biosynthesis | 0.23387 | 3.8025 |
| hsa00410 | beta-Alanine metabolism | 0.24408 | 3.6214 |
| hsa01240 | Biosynthesis of cofactors | 0.26644 | 1.6653 |
| hsa00010 | Glycolysis / Gluconeogenesis | 0.29321 | 2.925 |
| hsa00250 | Alanine, aspartate and glutamate metabolism | 0.312 | 2.7161 |
| hsa00590 | Arachidonic acid metabolism | 0.38253 | 2.1125 |
| hsa01040 | Biosynthesis of unsaturated fatty acids | 0.38253 | 2.1125 |
| hsa00520 | Amino sugar and nucleotide sugar metabolism | 0.39084 | 2.0554 |
| hsa00240 | Pyrimidine metabolism | 0.40716 | 1.95 |
| hsa01250 | Biosynthesis of nucleotide sugars | 0.42306 | 1.8549 |
| hsa00970 | Aminoacyl-tRNA biosynthesis | 0.47557 | 1.5844 |
| hsa01232 | Nucleotide metabolism | 0.51694 | 1.4083 |
| hsa00230 | Purine metabolism | 0.58485 | 1.17 |
